# The influence of recent and future climate change on spring Arctic cyclones

**DOI:** 10.1038/s41467-022-34126-7

**Published:** 2022-11-09

**Authors:** Chelsea L. Parker, Priscilla A. Mooney, Melinda A. Webster, Linette N. Boisvert

**Affiliations:** 1 Cryospheric Sciences Lab, NASA Goddard Space Flight Center, Greenbelt, MD 20771 USA; 2 Earth System Science Interdisciplinary Center, University of Maryland, College Park, MD 20740 USA; 3 NORCE Norwegian Research Centre, Bjerknes Centre for Climate Research, Jahnebakken 5, 5007 Bergen, Norway; 4 University of Alaska Fairbanks, Geophysical Institute, Fairbanks, AK 99775 USA

**Keywords:** Atmospheric dynamics, Climate and Earth system modelling, Cryospheric science

## Abstract

In recent decades, the Arctic has experienced rapid atmospheric warming and sea ice loss, with an ice-free Arctic projected by the end of this century. Cyclones are synoptic weather events that transport heat and moisture into the Arctic, and have complex impacts on sea ice, and the local and global climate. However, the effect of a changing climate on Arctic cyclone behavior remains poorly understood. This study uses high resolution (4 km), regional modeling techniques and downscaled global climate reconstructions and projections to examine how recent and future climatic changes alter cyclone behavior. Results suggest that recent climate change has not yet had an appreciable effect on Arctic cyclone characteristics. However, future sea ice loss and increasing surface temperatures drive large increases in the near-surface temperature gradient, sensible and latent heat fluxes, and convection during cyclones. The future climate can alter cyclone trajectories and increase and prolong intensity with greatly augmented wind speeds, temperatures, and precipitation. Such changes in cyclone characteristics could exacerbate sea ice loss and Arctic warming through positive feedbacks. The increasing extreme nature of these weather events has implications for local ecosystems, communities, and socio-economic activities.

## Abstract (web summary)

Rapid warming and Arctic sea ice melt will drive intensification of regional storms, increasing wind speeds and precipitation. Increasingly extreme storms exacerbate climate change effects and damage to local ecosystems, communities, and industries.

## Introduction

With increasing global average ocean and air temperatures and amplified Arctic warming in recent decades (e.g. refs. 1–4), Arctic sea ice has experienced a rapid decline in extent^5–8^, concentration^9^, thickness^10,11^, and depth of its snowpack^12^. Coincident with these declines, there has been a lengthening of the melt season^13,14^, and a transition from perennial ice to a seasonal ice^15–17^. There has also been a significant decline in winter sea ice growth, maximum extent, concentration, and thickness^11,18–20^. Reductions in winter sea ice have important feedback effects on atmospheric processes^21–23^ and may drive changes in Arctic cyclone intensification and behavior during winter and into spring^24,25^. Indeed, recent studies have shown that Arctic Eady growth rates (rapid increases in baroclinic disturbance) have significantly increased in winter and spring over the past four decades as low-level static stability in the atmosphere has decreased with retreating sea ice^8^. Interactions between the ice and atmosphere during the spring can be influential in determining sea ice survivability during the melt season^26,27^. While global models from the Climate Model Intercomparison Project (CMIP6) still exhibit large biases and a spread of projected Arctic change under different climate scenarios, there is strong agreement that Arctic sea ice loss and Arctic amplification will continue through this century^28^.

Arctic cyclones are low-pressure systems of varying size (~200–1500 km in radius) and are often associated with large changes in temperature and humidity, strong winds, and heavy persistent precipitation. Cyclones can act as compound extreme events (when extremes in two or more meteorological parameters, such as wind and precipitation, occur in tandem), exacerbating their negative impacts^29^. Extreme polar weather events are hazardous to local communities, maritime activities, and economically significant industries such as commercial fishing, shipping, and oil and gas retrieval^30,31^. Arctic cyclones typically track poleward and are therefore a major mechanism for heat and moisture transport to the Arctic from lower latitudes (e.g. ref. 32). Cyclone winds can influence sea ice motion^33–35^ and the thermodynamic characteristics can alter the surface energy budget, triggering sea ice growth or melt^27,36,37^. The interactions between cyclones, sea ice, and the local climate are complex because they vary with a given cyclone’s characteristics, location, duration, and season (e.g. refs. 38, 39). Interactions can result in either sea ice growth or loss depending on the net effect of factors during each event. Improving our understanding of Arctic cyclones and the contribution of climate change to their behavior is crucial for anticipating the effects of cyclones on sea ice mass balance, the freshwater budget, and the surface energy balance in the warming Arctic. Improved understanding and predictions of cyclone activity will become increasingly important for society in the coming decades as commercial and industrial development increases^40,41^ with continued sea ice retreat^42^.

The interplay between the global and local surface, oceanic, atmospheric, and flux changes, and Arctic cyclone behavior associated with climate change remain poorly understood. Some studies suggest that changes to the land–sea temperature contrast and thermal structure of the Arctic atmosphere may increase baroclinicity and subsequently cyclogenesis and cyclone activity^43–45^. Increases in surface turbulent fluxes with climate change^46^ may encourage overturning and deep convection, resulting in more intense cyclones^47^. However, observations are sparse at these locations and analyses of long-term trends in Arctic cyclone activity from observational and reanalysis data remain inconclusive. Studies of recent decades have revealed substantial spatio-temporal variability in the analysis of cyclone frequency and characteristic changes^24,38,48–53^.

While some studies have investigated the possible broad-scale impacts of future climate change on Arctic cyclones, few have examined the contribution of recent climate change to cyclone dynamics. Global models generally agree in predicting increased summer cyclone activity by the end of the 21st Century^54–57^. However, they largely disagree in their predictions of winter cyclone activity^57–59^. The discrepancies may be symptomatic of the spatial resolution of global models, which are too coarse to accurately represent pertinent local scale processes^60^ and cyclone characteristics^55,57,61^. To advance our understanding of how recent climate change has influenced Arctic cyclone events and explore how cyclone behavior may be altered with continued warming and sea ice loss, it is imperative to use techniques that can realistically simulate Arctic atmospheric processes and weather events at the kilometer scale.

In this study, we examine the influence of both recent and future climate change on major characteristics of Arctic cyclones during spring (March and April). Spring represents an important period for sea ice–atmosphere interactions, where the changing sea ice conditions may affect cyclone development and characteristics, and top-down forcings from cyclone activity could precondition the sea ice for the summer melt season. Case studies were found using a Lagrangian cyclone finding and tracking scheme (see the “Methods” section) and the primary case studies presented here coincided with the 2019–2020 Multidisciplinary drifting Observatory for the Study of Arctic Climate (MOSAiC) expedition^62^ for high-resolution validation and insights. Cyclones are simulated using a regional configuration of the National Center for Atmospheric Research (NCAR) Weather Research and Forecasting (WRF) model^63^ at convection-permitting resolutions. High spatial (≤4 km) and temporal (sub-hourly) resolutions are crucial for capturing cyclone intensification and decay and the complex interactions with the local environment. The current climate simulations provide detailed insight into cyclone lifecycles and their characteristics during the spring. The contributions of climate change to extreme events are addressed using the “Storylines” approach^64^ and a Pseudo Global Warming (PGW) technique. Large-scale climate perturbations are derived from the Climate Model Intercomparison Project (CMIP6)^65^ and downscaled onto the regional modeling framework to simulate the events under historical and future climate perturbations. This approach allows us to improve our understanding of Arctic cyclones and climate interactions at the process level by isolating the storm-scale changes arising from changes in the large-scale dynamic and thermodynamic environment. The results address whether sufficient sea ice decline and atmospheric warming and moistening have already occurred to influence spring Arctic cyclone behavior to date and whether continued ice decline and environmental changes will alter cyclone intensification, trajectories, and characteristics by the end of this century.

## Results

### Climate change causes Arctic sea ice loss, atmospheric warming, and changes to humidity and wind patterns

**Fig. 1:**
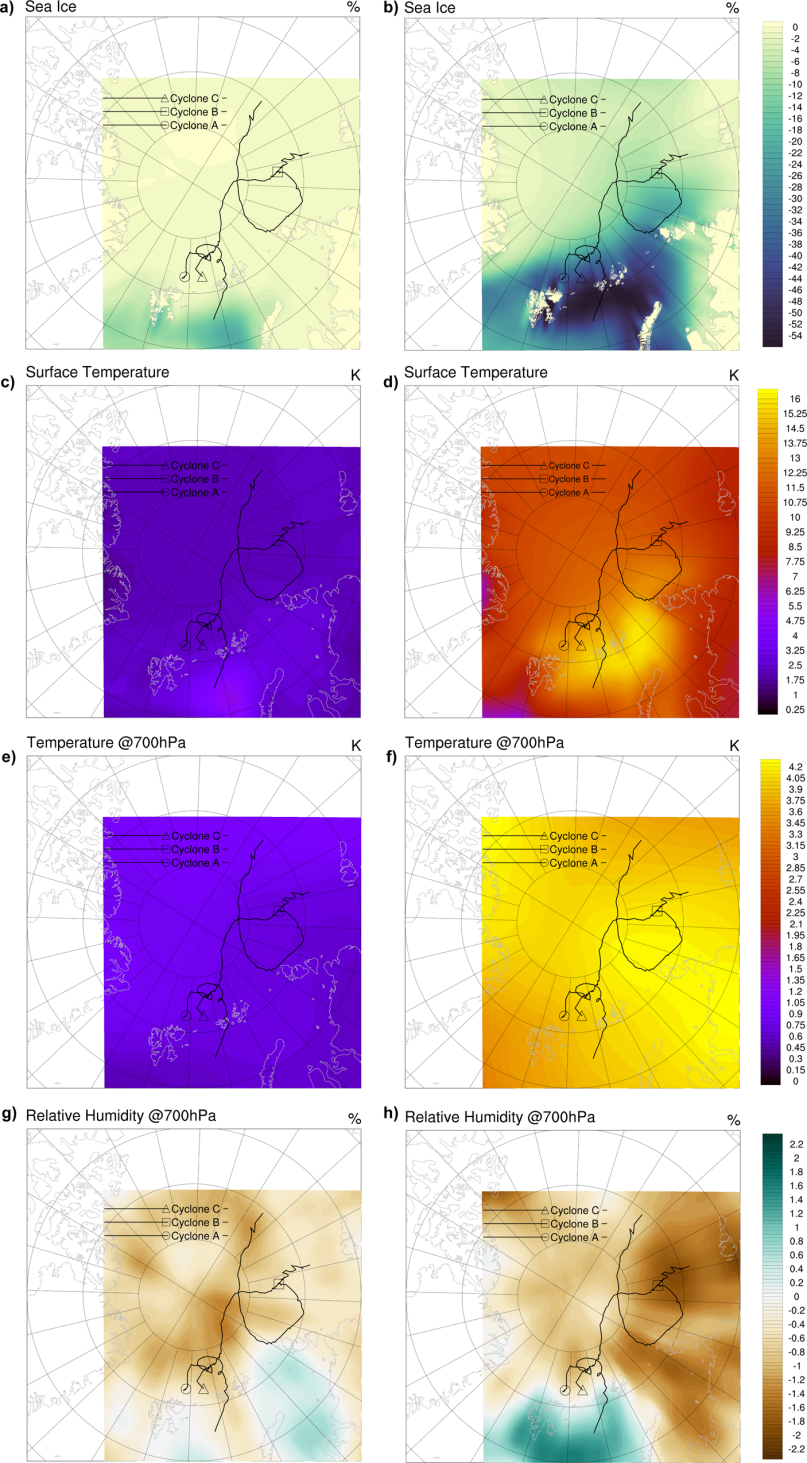
The historical and future March climate change deltas calculated from Climate Model Intercomparison Project 6 results. The calculated difference in March sea ice concentration, surface temperature, and temperature and relative humidity at 700 hPa for the average current (1985–2014) minus the average historical (1886–1915) climate (panels **a**, **c**, **e**, **g**, respectively) and the average future (2070–2100) minus the average current climate (panels **b**, **d**, **f**, **h**). The simulated tracks of cyclones A–C in the current climate are overlain. Markers show the end of the trajectories.

**Fig. 2:**
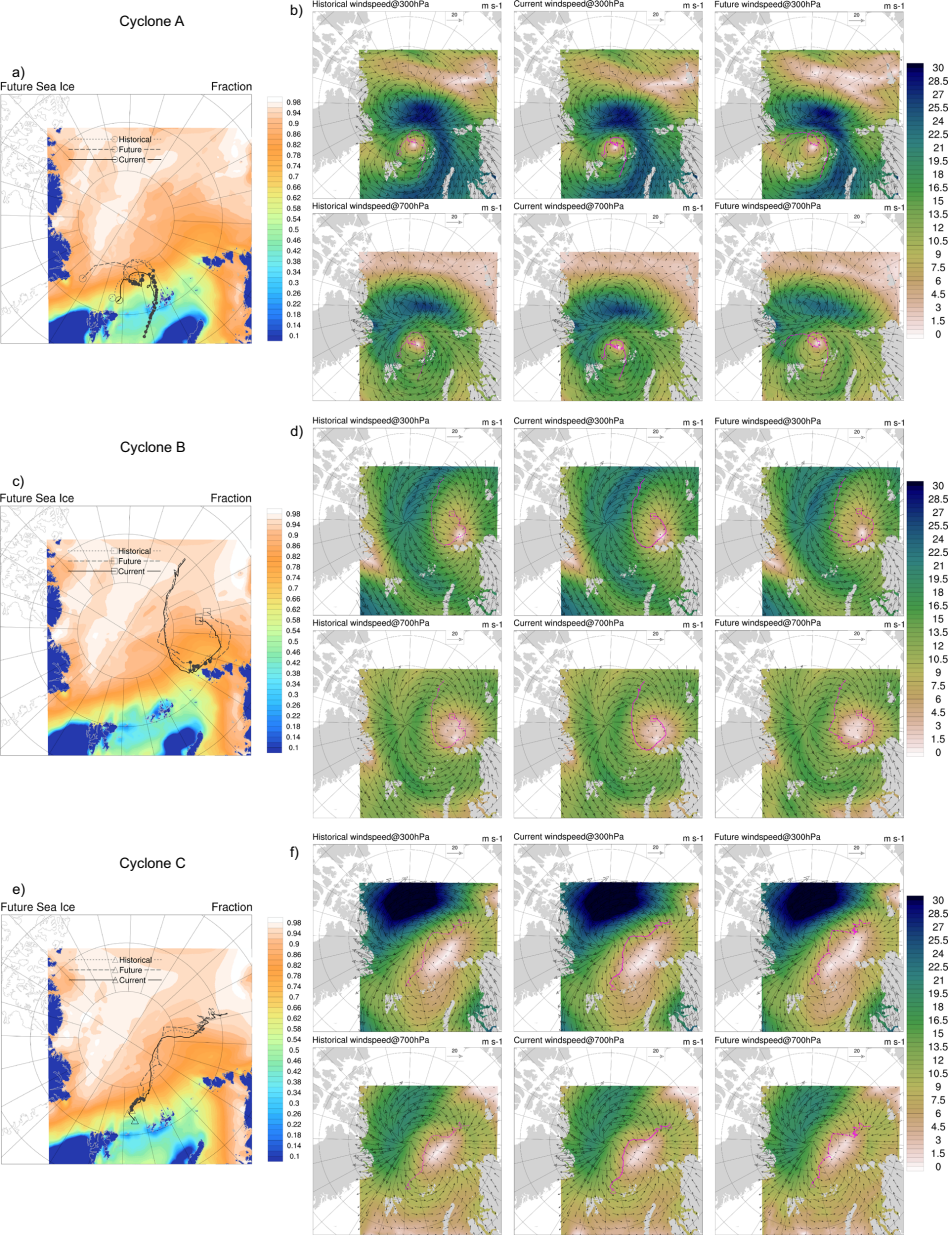
Alterations in upper-level atmospheric steering flow and cyclone trajectories in response to climate perturbations. Panels **a**, **c**, **e** show the average sea ice concentration (fraction) in the future climate simulations of Cyclones A–C respectively, with the cyclone tracks from historical, current, and future climate simulations overlain. Open markers show the end of the trajectories, filled markers indicate where the average sea ice concentration beneath the cyclone is ≤75%. Panels **b**, **d**, **f** show the average wind speeds (m s^−1^) and vectors at 700 and 300 hPa over the historical, current, and future climate simulations with the resulting cyclone track overlain for each case study.

The differences between the average current (1985–2014) and historical (1885–1914) climate from CMIP6 models show that during March and April, the region experiences a ~0–25% decrease in sea ice concentration (Fig. 1a; Supplementary Fig. 2a) and a ~1.5–6.5 °C increase in surface temperatures since 1914 (Fig. 1c; Supplementary Fig. 2c). The most notable changes are seen in the Barents Sea region. Aloft in the atmosphere, there are relatively small increases in temperature of ~0.5–1.4 °C at 700 hPa (Fig. 1e; Supplementary Fig. 2e) with higher relative humidity (~0.5%) over portions of the Barents and Kara Seas and lower humidity elsewhere in the domain (~0.4–1.5%, Fig. 1g; Supplementary Fig. 2g). The winds higher up in the atmosphere (steering flow), act to direct atmospheric circulation and weather patterns and therefore influence cyclone trajectories in the Arctic. However, there are only minimal differences in the large-scale, steering flow from the historical to current climates (Fig. 2b, d, f).

The differences between the average March future (2070–2099) and current (1985–2014) climate are more pronounced over the study region. By the end of the 21st century, sea ice concentration is predicted to decrease up to ~60% (Fig. 1b; Supplementary Fig. 2b), with subsequent increases in surface temperatures by ~4–17 °C (Fig. 1d; Supplementary Fig. 2d). The greatest change in surface conditions is in the northern Barents and Kara seas towards the central Arctic. The increasing temperatures propagate up through the atmosphere, with increases of ~3.5–4.4 °C at 700 hPa (Fig. 1f; Supplementary Fig. 2f). There is higher relative humidity over the Greenland and Barents Seas (~0.5–1.5%) but lower relative humidity throughout the rest of the domain (~0–2.5%, Fig. 1h; Supplementary Fig. 2h). In the current climate, there is a distinct range in the strength and location of the anticyclone blocking high, the structure and positioning of the cyclonic flow, and therefore the overall steering flow throughout the spring (Fig. 2b, d, f). In the future climate scenario, there is a slight weakening and geographic shift in the anticyclonic blocking high and the cyclonic flow, resulting in an alteration of the atmospheric steering flow (Fig. 2b, d, f).

### The modeling framework realistically represents cyclone characteristics in the current climate

**Fig. 3:**
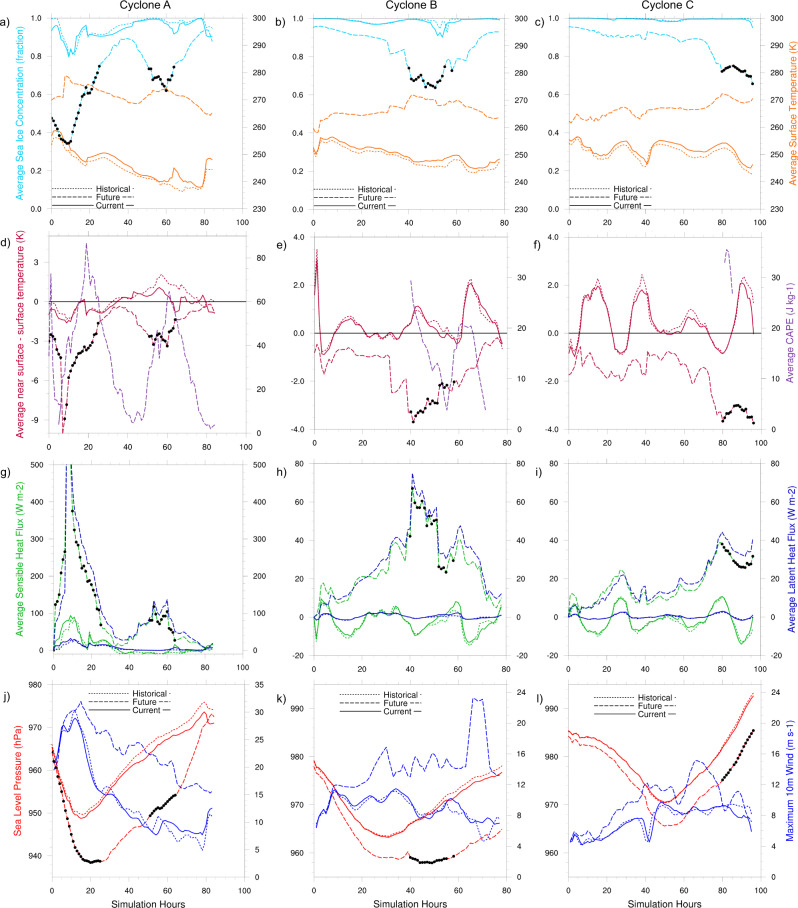
The evolution of surface and thermodynamic conditions and cyclone intensity over the cyclone lifecycles in contrasting climates. The hourly sea ice concentration (fraction) and surface temperature (K) (**a**–**c**), the difference in near-surface air temperature and surface temperature (K) and Convective Available Potential Energy (J kg^−1^) (**d**–**f**), sensible and latent heat fluxes (W m^−2^) (**g**–**i**) averaged over a 200 km^2^ area around the cyclone center point throughout the simulations. Panels **j**–**l** show the sea level pressure (hPa) at the center-point of the cyclone and the maximum 10 m wind speed in a 200 km^2^ area around the storm center. The black markers indicate time steps where the average sea ice concentration in the same area is ≤75%.

The current climate cyclones predominantly track over areas with high sea ice coverage (~100% concentration). However, some cases spend portions of their lifecycle over areas of lower average sea-ice concentration at lower latitudes or in the peripheral seas, e.g., Cyclone A where the average sea ice concentration is ~80% in the Barents Sea and Fram Strait areas (Fig. 3a–c). At higher latitudes, surface temperatures in the current climate are below freezing (from ~−37 to −15 °C, Fig. 3a–c; Supplementary Figs. 6a–c, 7a–c) and the near-surface (2 m) air temperature is either similar or up to 2 °C warmer (Fig. 3d–f; Supplementary Figs. 6d–f, 7d–f), resulting in sensible heat fluxes fluctuating around zero (Fig. 3g–i; Supplementary Figs. 6g–i, 7g–i). Given that a consolidated sea ice pack inhibits heat exchange between the atmosphere and ocean, there is minimal latent heat flux (Fig. 3g–i; Supplementary Figs. 6g–i, 7g–i). The directionality and magnitude of the energy fluxes vary with the vertical temperature gradient between the surface and atmosphere (Fig. 3d–f; Supplementary Figs. 6d–f, 7d–f). The environment supports the intensification of cyclones with minimum sea level pressures (SLP) of ~949–979 hPa and maximum near-surface wind speeds of ~5–29 m s^−1^ (Fig. 3j–l; Supplementary Figs. 6j–l, 7j–l). For all cases, the cyclones originate at lower latitudes and their individual tracks into higher latitudes are governed by the location, strength, and structure of the anticyclonic, high pressure and cyclonic, low-pressure circulation during the time of the event (Fig. 2b, d, f; Supplementary Figs. 4, 5). Detailed comparisons of model simulations (Cyclones A–C) and in situ data from the MOSAiC expedition demonstrate that the model configuration developed for this study can accurately represent atmospheric variables and their change over time during cyclone events (see the “Methods” section and Supplementary Fig. 9). We use these current climate results as a baseline to assess the relative changes in cyclone behavior and characteristics in the historical and future climate perturbation simulations.

### Recent climate change has not appreciably affected spring Arctic cyclone characteristics

**Fig. 4:**
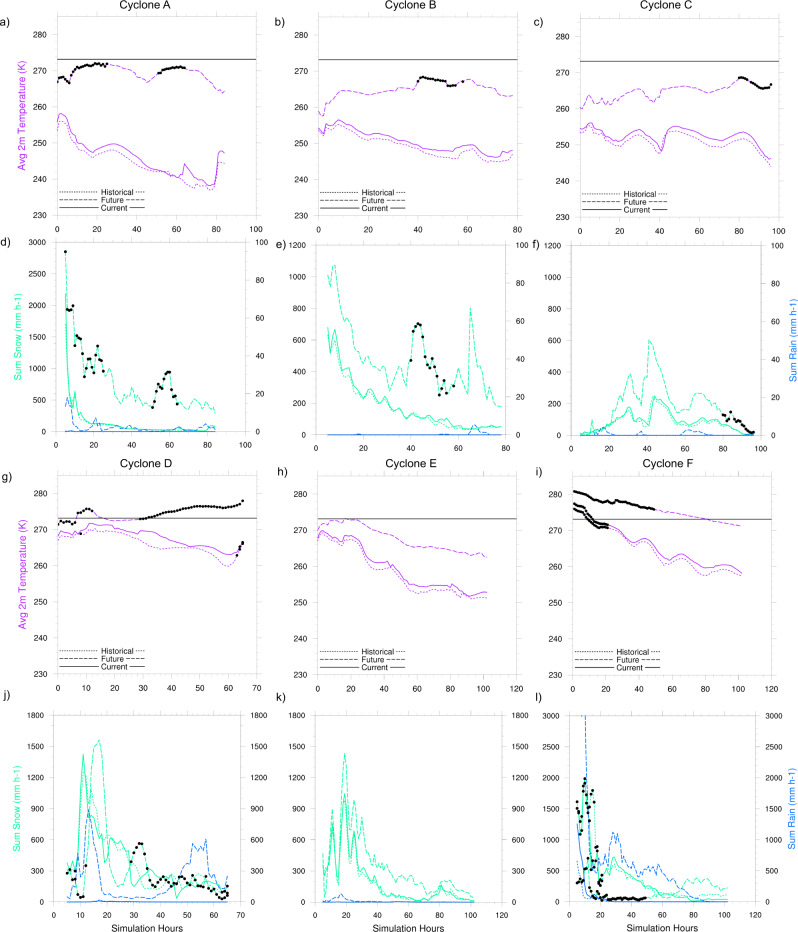
The evolution of near-surface air temperature and precipitation (snow and rain) rates over cyclone lifecycles in contrasting climates. The hourly cyclone near surface (2 m) temperature (K) averaged and snowfall and rainfall rates (mm h^−1^) summed over a 200 km^2^ area around the cyclone center throughout the simulations for March Cyclones A–C (panels **a**–**f**) and April Cyclones D–F (panels **g**–**l**). Note that for March simulations, rainfall is on a different scale to snowfall (panels **d**–**f**). The black markers indicate time steps where the average sea ice concentration in the same area is ≤75%. The solid line in panels **a**–**c**, **g**–**i** marks the melting/freezing point (0 °C).

The changes in surface and atmospheric conditions with recent climate change have not yet been sufficient to significantly alter cyclone characteristics and behavior in the spring. In the historical climate, the cyclones experience slightly greater sea ice concentrations (if not already at 100% in the current climate) and lower average surface temperatures than in the current climate (Figs. 1a–c and 3a–c; Table 1; Supplementary Figs. 6a–c, 7a–c; Supplementary Tables 3, 4). The temperature changes result in only minor increases in the air-surface temperature difference (Fig. 3d–f; Table 1; Supplementary Figs. 6d–f, 7d–f; Supplementary Tables 3, 4) and thus minimal changes in the direction or magnitude of the surface turbulent fluxes (Fig. 3g–i; Table 1; Supplementary Figs. 6g–i, 7g–i; Supplementary Tables 3, 4). The cyclone intensities and lifecycles remain largely unchanged between the historical and current climate. However, Cyclones A, B, D, and I undergo more weakening during the decay phase in the historical climate (Fig. 3j, k; Supplementary Figs. 6j, 7k). The weakening occurs when sea ice concentration is greater in the history than the current climate and the air temperature exceeds the surface temperature, directing the sensible heat flux from the atmosphere to the surface (Fig. 3; Supplementary Figs. 6, 7). Recent climate change has not systematically altered the cyclone size (median radius, Table 1, Supplementary Tables 3, 4), nor has it substantially changed the precipitation rates, despite slight increases in cyclone near-surface air temperatures (Fig. 4; Table 1; Supplementary Fig. 8; Supplementary Tables 3, 4). Importantly, the results demonstrate a consistent cyclone response to the historical climate forcing across all cases. The relative change in cyclone characteristics is robust even when considering cases of differing years from March (Cyclones A, B, C, G, H, I) or cases from April (Cyclone D, E, F). Note that an April climate change delta is applied to the April cases (see Supplementary Fig. 2). Accordingly, the simulation results suggest that recent surface and thermodynamic changes have had a negligible effect on spring cyclone intensification and characteristics.

**Table 1:** Numerical summary of changes in simulated environmental and cyclone characteristics (Cases A–C) from historical and future climate perturbation

Change in characteristic	Cyclone A CC-HC	Cyclone B CC-HC	Cyclone C CC-HC	Cyclone A FC-CC	Cyclone B FC-CC	Cyclone C FC-CC
Average sea ice concentration (%)	Max: −6.19	Max: −8.32	Max: −3.26	Max: −54.45	Max: −34.36	Max: −29.43
	Avg: −1.41	Avg: −0.47	Avg: −0.17	Avg: −22.31	Avg: −14.32	Avg: −12.42
Average surface temperature (°C)	Max: 7.34	Max: 3.18	Max: 3.55	Max: 34.39	Max: 24.37	Max: 24.31
	Avg: 2.00	Avg: 1.44	Avg: 1.53	Avg: 25.43	Avg: 16.23	Avg: 14.52
Minimum sea level pressure (hPa)	Max: −3.56	Max: −1.44	Max: −0.82	Max: −15.26	Max: −12.90	Max: −7.37
	Avg: −1.43	Avg: −0.43	Avg: −0.07	Avg: −10.41	Avg: −7.37	Avg: −4.23
% Lifecycle at maximum intensity	0	1.27	0	5.88	24.05	−2.06
Maximum 10 m wind speed (m s^−1^)	Max: 3.63	Max: 2.99	Max: 2.13	Max: 15.22	Max: 16.94	Max: 7.23
	Avg: −0.02	Avg: 0.24	Avg: −0.16	Avg: 8.33	Avg: 5.05	Avg: 2.46
Average sensible heat flux (W m^−2^)	Max: 28.68	Max: 5.02	Max: 5.56	Max: 524.80	Max: 74.37	Max: 40.16
	Avg: 3.73	Avg: 0.52	Avg: 0.51	Avg: 88.43	Avg: 28.12	Avg: 18.33
Average latent heat flux (W m^−2^)	Max: 11.06	Max: 1.65	Max: 1.05	Max: 543.63	Max: 75.56	Max: 41.26
	Avg: 1.80	Avg: 0.17	Avg: 0.14	Avg: 107.54	Avg: 28.74	Avg: 18.07
Average 2 m temp.—surface temp.	Max: −2.09	Max: −0.42	Max: −1.27	Max: −8.64	Max: −4.55	Max: −5.28
	Avg: −0.38	Avg: −0.04	Avg: −0.12	Avg: −2.09	Avg: −1.90	Avg: −2.25
Average CAPE (J kg^−1^)	Max: 12.75	Max: 0	Max: 0	Max: 86.70	Max: 29.35	Max: 35.52
	Avg: 0.20	Avg: 0	Avg: 0	Avg: 33.27	Avg: 6.14	Avg: 1.67
Average 2 m temp. (°C)	Max: 5.25	Max: 3.00	Max: 2.40	Max: 30.55	Max: 19.83	Max: 20.49
	Avg: 1.62	Avg: 1.40	Avg: 1.41	Avg: 23.39	Avg: 14.33	Avg: 12.27
Sum snowfall in 200 km area (mm h^−1^)	Max: 480.38	Max: 70.28	Max: 41.80	Max: 1624.57	Max: 764.27	Max: 544.88
	Avg: 18.10	Avg: 14.08	Avg: 8.06	Avg: 642.77	Avg: 293.99	Avg: 104.23
Sum rainfall in 200 km area (mm h^−1^)	Max: 0.27	Max: 0	Max: 0	Max: 17.83	Max: 5.22	Max: 4.18
	Avg: 0.01	Avg: 0	Avg: 0	Avg: 2.27	Avg: 0.31	Avg: 0.59
Median radius (km)	Max: 145.93	Max: 279.21	Max: 171.45	Max: 314.92	Max: 193.76	Max: 270.97
	Avg: −19.88	Avg: 14.72	Avg: −8.62	Avg: 24.93	Avg: −47.16	Avg: 60.49

The average and the maximum difference in the surface, atmospheric, near-surface flux, and cyclone variables of interest over the length of the simulations. Differences are calculated as (current climate (CC)−historical climate (HC)) and (future climate (FC)−current climate (CC)) simulation at each hourly timestep for each cyclone case A–C.

The simulations also show that the dynamic, large-scale climate perturbation from recent climate change is generally small and has no appreciable effect on cyclone tracks in this season (Fig. 2; Supplementary Figs. 4, 5), with the exception of Cyclone A. For Cyclone A under the historical climate perturbation, there is a slight weakening and poleward shift of the steering flow that results in a poleward deviation in the trajectory (Fig. 2a, b).

Collectively, these differences in cyclone characteristics between the current and historical climates cannot be separated from model internal variability and are therefore not significant. Therefore, despite declining sea ice, increasing surface temperature, and atmospheric changes since the industrial revolution (see Fig. 1; Supplementary Figs. 2, 3), cyclone lifecycles and characteristics during spring have not yet been notably altered due to recent climate change.

### Future climate change increases and prolongs cyclone intensity

Unlike the effects of recent climate change, future climate conditions act to increase the surface-air temperature gradients, surface fluxes into the atmosphere, and increase and prolong cyclone intensity. Climate models project that with continued anthropogenic climate change and Arctic Amplification, there will be significant sea ice loss and increased surface and atmospheric temperatures by the end of the 21st century (shown by the climate delta in Fig. 1b, d, f; Supplementary Fig. 2b, d, f). With future climate change, cyclones travel over areas of substantially reduced sea ice concentration and higher surface temperatures (Fig. 1b, d, f, Supplementary Figs. 2b, d, f, 3b, d, f; Fig. 2a, c, e; Supplementary Figs. 4, 5; Fig. 3a–c, Supplementary Figs. 6a–c, 7a–c). The climate change deltas and simulation results show that there is considerable warming throughout the environment (Fig. 1d, f; Supplementary Fig. 2d, f). However, the surface is warming more rapidly than the atmosphere, driving large changes in the temperature gradient between the atmosphere and surface (Fig. 3d–f, Table 1; Supplementary Figs. 6d–f, 7d–f, Supplementary Tables 3, 4). The combination of the increase in the vertical temperature gradient, moisture availability from the increasingly exposed ocean surface, and the potential for the atmosphere to hold more water vapor, results in a large change in the magnitude and direction of the surface turbulent fluxes (sensible and latent heat). These fluxes increase on average by ~4–108 W m^−2^ and are transferred from the surface to the atmosphere during the cyclone lifecycles in the future climate (Fig. 3g–i, Table 1; Supplementary Figs. 6g–i, 7g–i; Supplementary Tables 3, 4). The largest increases in air-surface temperature difference and positive surface turbulent fluxes into the atmosphere clearly occur when the average sea ice concentration under the cyclone is <75% (Fig. 2a, c, e, Fig. 3, Supplementary Figs. 4–7 black, circle markers). The changes to the surface and atmosphere support the development of convective available potential energy (CAPE) in the atmosphere which is not induced in the current or historical climates (Fig. 3d–f, Table 1; Supplementary Figs. 6d–f, 7d–f; Supplementary Tables 3, 4). The presence of CAPE indicates instability in the atmosphere and the amount of energy available for updrafts, convection, and overturning in the atmosphere. While the results do not show a consistent response in cyclone size (Table 1; Supplementary Tables 3, 4), the thermodynamic changes result in increased and prolonged cyclone intensity and enhanced wind speeds of 1.5–8 m s^−1^ on average but up to 17 m s^−1^ at certain time steps (Fig. 3j–l, Table 1; Supplementary Figs. 6j–l, 7j–l; Supplementary Tables 3, 4)). Minimum sea level pressure (SLP) decreases on average by ~4–12 hPa (up to 28 hPa), and the percentage of the cyclone lifecycles spent at maximum intensity (within 5 hPa of the minimum) is typically prolonged by 1.5–31% (Table 1, Supplementary Tables 3, 4). The relative change in cyclone characteristics is consistent even when examining cases across different years and from both March (Cyclone A–C, G–I) or April (Cyclone D–F), with March or April climate change delta applied, respectively (Fig. 1, Supplementary Figs. 2, 3). Therefore, the simulations reveal that surface energy fluxes, deep convection, and wind speeds increase with cyclone intensity as sea ice declines and surface temperatures increase in the future climate.

Increases in surface turbulent fluxes and moisture into the atmosphere during cyclone activity in winter and spring contribute to further atmospheric warming and Arctic Amplification^23^. The warming promotes further sea ice decline in a positive feedback^66,67^. Increasing wind speeds with future cyclone activity could drive more dynamic sea ice motion and ice thickness redistribution^68,69^. Enhanced wind activity and growing lead areas from sea ice divergence will result in increased upper ocean mixing and energy transfer from the ocean to the atmosphere^70^. Augmented energy transfer could act to increase cyclone intensities with positive feedback. The greater presence of CAPE increases and prolongs cyclone intensity and delays decay during cyclone events (Fig. 3j–l; Table 1; Supplementary Figs. 6j–l, 7j–l; Supplementary Tables 3, 4). Therefore, the exacerbated cyclone conditions may affect a greater area of sea ice and ocean and other components of the Arctic system for a longer duration in a warmer climate.

### Cyclone precipitation and temperature increase with future climate change

Surface and thermodynamic changes with future climate change result in a substantial increase in precipitation and air temperature during cyclone events. Enhanced cyclone precipitation is fueled by increasing moisture availability with declining sea ice cover, increasing latent heat fluxes from the surface into the atmosphere, and the ability of the atmosphere to hold more water vapor with increasing temperatures. Despite the large increases in surface and atmospheric temperatures at all levels by the end of the 21st century (Fig. 1d, f; Supplementary Fig. 3d, f), the simulations show that the augmented cyclone-associated precipitation still falls primarily as snow in March where the sum of snowfall in a 200 km^2^ area around the cyclone center increases on average by ~94–643 mm h^−1^ (Fig. 4d–f; Table 1; Supplementary Fig. 8d–f; Supplementary Table 4). The enhancement of snowfall found here is in line with predicted spring precipitation changes by the end of the century in other studies (e.g. refs. 71–73). An increase in cyclone-associated snowfall in spring would act to thicken the snowpack over sea ice as solar insolation increases with the polar day. A thicker snowpack requires more energy to melt and thus may help delay sea-ice surface melt in late spring and early summer^74,75^. Even so, the simulations show that near-surface air temperatures during March cyclones increase on average by ~11–23 °C, pushing the air temperatures closer to the freezing/melting point with the future climate perturbation (Fig. 4a–c; Table 1; Supplementary Fig. 8d–f; Supplementary Table 4). Warmer temperatures and increased longwave downwelling radiation with cyclones may result in snow and sea-ice melt^27,76,77^. Results demonstrate that the temperature increase facilitates more mixed-phase and liquid precipitation. Small amounts of rain precipitate over sea ice during March cyclones even at high latitudes as air temperatures approach the freezing threshold in the future climate scenario (Fig. 4; Table 1; Supplementary Fig. 8; Supplementary Table 4). Furthermore, the future climate simulations reveal that spring cyclone air temperatures can increase above the freezing point at lower latitudes and when solar insolation begins to increase later into the season (April) (Fig. 4g–i; Supplementary Fig. 8a–c). Under these conditions, we see a distinct phase change in precipitation and the sum of rain around the cyclone center increases on average by ~13–846 mm h^−1^ in the future climate, from near zero in the current and historical climate (Fig. 4j–l; Supplementary Fig. 8d–f; Supplementary Tables 3, 4). Both increased temperatures above freezing and rain on snow would act to coarsen the uppermost snow grains of the snowpack, which may reduce the reflectivity of the snow cover^78^ and lead to greater snow and sea-ice melt in spring and summer. Cases D and F demonstrate that during events where near-surface air temperatures are above freezing for most of the cyclone lifecycle, there is an overall decrease in snowfall with future climate change (Fig. 4j–l; Supplementary Table 3). A reduction in fresh snow accumulation would reduce snowpack depth, requiring less energy to melt and potentially altering the onset and rate of sea ice loss during the melt season. The net effect of precipitation and temperature changes on snow and sea-ice mass balance is complex and may vary with each cyclone event and sea-ice conditions in the future climate.

### Future Arctic cyclones may impact previously unaffected areas

The climate delta calculations suggest that the thermodynamic changes over the Arctic will be accompanied by slight changes to the large-scale atmospheric dynamics by the end of the century (Fig. 2b, d, f). The high-resolution simulations show that cyclone trajectories are strongly influenced by large-scale atmospheric flow even as the cyclones increase in intensity. Future climate change alters the steering flow by slightly weakening the anticyclonic blocking high and shifting the structure and positioning of the cyclonic circulation (Fig. 2). Results show that this type of perturbation will alter future cyclone trajectories, potentially increasing their geographic range. However, the extent of the large-scale future dynamical change and the subsequent effect on the steering flow and cyclone trajectory differs for each case. For example, during the lifecycle of Cyclone A, the anticyclone blocking high weakens and shifts further poleward and eastward while the cyclonic circulation weakens and widens (Fig. 2b). These changes allow Cyclone A to travel further poleward and westward across the Central Arctic towards the Fram Strait in the future climate (Fig. 2a, b). During the lifecycle of Cyclone B in the future climate (Fig. 2d), there are only minor deviations in the large-scale flow causing the cyclone to drift slightly further poleward and then southward to the lower latitudes before tracking eastward back towards the pole as the cyclone maintains intensity (Figs. 2c, d and 3k). For Cyclone C, the strong steering flow of the high-pressure area governs the overall direction and poleward extent of the cyclone’s journey (Fig. 2f). With future climate perturbation, there is a weakening of the blocking high and the cyclonic circulation between the Laptev and East Siberian Seas towards the Central Arctic, allowing the cyclone to track further poleward and eastward early in the lifecycle. The slight weakening and shift of the cyclonic circulation around Franz Josef Land allow the cyclone to drift slightly further poleward and westward before heading towards the Barents Sea at the end of its lifecycle. Collectively, the results suggest that the geographical extent of the cyclone trajectories increases with future climate change (Fig. 2a, c, e; Supplementary Figs. 4, 5). Consequently, future track changes may bring increased and prolonged cyclone wind speeds, surface turbulent fluxes, air temperatures, and precipitation to areas that were previously not affected in the current or historical climates (e.g., areas closer to the north pole).

## Discussion

This study uses regional climate modeling techniques at convection-permitting scales (km-scale) with downscaled climate reconstructions and projections to answer the following questions: First, to what extent has recent climate change contributed to present-day spring cyclone characteristics in the Arctic? Second, how will continued climate change and Arctic Amplification affect spring cyclone behavior in the future?

The results demonstrate that even though recent climate change is altering the Arctic climate, cyclone characteristics do not yet exhibit an appreciable response in spring. While temperatures have increased, the absolute values remain below freezing in the current climate, and while sea ice characteristics have changed, large changes in concentration are confined to the marginal seas during spring. The minimal changes in the vertical air-surface temperature gradients and fluxes result in muted differences between the cyclone characteristics in the current and historical climates. However, with future projected climate change, substantial sea ice loss and increasing surface temperatures drive large changes in the vertical temperature gradient, sensible and latent heat fluxes, and convection during cyclone activity. The future thermodynamic changes increase and prolong cyclone intensity, wind speeds, temperatures, and precipitation rates. As cyclone air temperatures increase above freezing, the precipitation phase changes to rainfall in place of snowfall with important implications for sea ice mass balance and survivability during the melt season. Results indicate that cyclone trajectories are strongly governed by large-scale, upper-level atmospheric steering flow even as the cyclones increase in intensity. Therefore, future climate change perturbation to the upper-level winds will alter cyclone trajectories, possibly increasing their geographic extents and steering them and their effects towards previously unaffected areas.

This study highlights that cyclones are highly sensitive to sea ice and atmospheric conditions in the Arctic, and changes in cyclone characteristics are most pronounced over areas with the greatest change in sea ice concentration. Therefore, current efforts to limit climate change and sea ice loss are paramount for mitigating the impacts of extreme polar weather in regions of significant ice loss, such as the Barents, Bering, and Chukchi seas. The projected changes to cyclone characteristics have several implications for the future of the local Arctic and global climate. Snowpack thickening from increased snowfall rates during spring cyclone activity could delay sea-ice surface melt. However, decreasing sea ice cover and a shift in cyclone precipitation phase to rainfall with increasing temperatures could negate the positive effect of enhanced spring snow accumulation on sea ice mass balance. Increasing wind speeds, ocean mixing, heat and moisture fluxes, and air temperatures during cyclone activity could exacerbate atmospheric warming and ice loss and therefore increase cyclone intensity in a positive feedback cycle with climate change. These changes in cyclone characteristics may increase the compound nature (extremes in two or more weather variables) of these events and augment climate stresses on fragile Arctic ecosystems and coastal communities. Longer-lived cyclones with increased wind speeds and heavier precipitation may pose a particular hazard for commercial and industrial activities such as fishing, shipping, and oil and gas retrieval.

While this study has examined the cyclone response to recent and future climate change in spring (March and April), similar future changes in cyclone characteristics may occur in other seasons based on our findings of the relationship between sea-ice loss and cyclone intensification. Sea ice concentration is predicted to decrease in all seasons across the Arctic with concurrent increases in temperature and moisture availability in a warming climate. The findings of this paper prompt further detailed analyses of the response of cyclone activity to climate change in contrasting seasons and climate scenarios.

## Methods

### WRF Model set-up

All simulations in this study use the Weather and Research Forecasting (WRF) model v3.9.1.1^64^ over the domain shown in Supplemental Fig. 1 which has a horizontal grid spacing of ~4 km. The model deploys 51 vertical levels up to 10 hPa (model top) with increased resolution in the planetary boundary layer. The WRF model configuration is informed by settings used in the previous studies^79–81^ and model configuration testing. The following WRF physical parameterizations are chosen based on their suitability for Arctic cyclone simulations at these convection-permitting scales: Rapid Radiative Transfer Model for General circulation models (RRTMG) shortwave and longwave radiation schemes^82^, Eta similarity surface layer scheme^83^, Unified Noah Land Surface Model^84^ for land surface processes and structure, the Mellor-Yamada-Janjic planetary boundary layer scheme^83^, the Morrison 2-moment microphysics scheme^85^. Differences in cloud microphysical parameterizations contribute to inter-model spread in the representation of the Arctic cloud annual cycle^86^. Schemes that treat cloud ice and liquid condensate uniquely, as separate prognostic variables, reduce biases in simulated cloud processes in the Arctic region^86^. Therefore, we chose the Morrison scheme which is a double-moment, 6-class scheme that predicts the number concentrations and mixing ratios for cloud droplets, cloud ice, snow, rain, and graupel independently. Other WRF settings of particular importance to the polar regions include daily updates of fractional sea ice concentration (SIC) and sea surface temperatures (SSTs) at 6-h intervals.

All simulations are initialized shortly after the apparent cyclone genesis time from the cyclone tracking algorithm and the simulation length captures the main development, intensification, and decay periods of the cyclone’s lifecycle. The start date and integration period for each simulation are shown in Table 2 and Table S2. All simulations use 3 h of the WRF digital filter initialization (DFI) procedure^87,88^ to fully balance the mass and wind fields at the initial time. This procedure reduces noise and imbalances created by perturbations to the model system^89^.

**Table 2:** Experimental set-up for the model simulations of cyclone case studies under different climate forcings

Run name	Initialization time (UTC)	Total integration time (h)	Climate conditions	CO_2_/CH_4_/N_2_O concentration (ppm/ppb/ppb)
CycloneA_CC	2020-03-12 00:00	90	ERA5 current climate	379/1774/319
CycloneB_CC	2020-03-18 18:00	78	ERA5 current climate	379/1774/319
CycloneC_CC	2020-03-30 00:00	96	ERA5 current climate	379/1774/319
CycloneA_HC	2020-03-12 00:00	90	ERA5 current climate + ΔHC	297/895/280
CycloneB_HC	2020-03-18 18:00	78	ERA5 current climate + ΔHC	297/895/280
CycloneC_HC	2020-03-30 00:00	96	ERA5 current climate + ΔHC	297/895/280
CycloneA_FC	2020-03-12 00:00	90	ERA5 current climate + ΔFC	975/2595/385
CycloneB_FC	2020-03-18 18:00	78	ERA5 current climate + ΔFC	975/2595/385
CycloneC_FC	2020-03-30 00:00	96	ERA5 current climate + ΔFC	975/2595/385

Details of the initialization time, duration, boundary conditions, and prescribed concentration of greenhouse gases for each of the cyclone case studies (A–C) and climate scenarios: current climate (CC), historical climate (HC), future climate (FC).

For current climate simulations, both boundary and initial conditions are derived from European Centre for Medium-range Weather Forecasts (ECMWF) Reanalysis 5th Generation data (ERA5)^90^, obtained on a 0.25° latitude × 0.25° longitude grid at 6-h intervals. Boundary and initial conditions for future and historical simulations are developed by perturbing these current climate boundary conditions following the pseudo-global warming (PGW) technique^91^.

### Pseudo-global warming perturbations

Perturbations are applied to the boundary and initial conditions of the current climate simulations to represent the historical climatic conditions of 1885–1914, and 2070–2099 under a future climate scenario. The advantage of this approach is that it isolates the response of cyclone characteristics to changes in the large-scale climate system. The PGW approach has previously been used to explore the response of weather systems (e.g., tropical cyclones, medicanes, and mid-latitude heavy rainfall events) to projected future climate change (e.g. refs. 92–96) and recent anthropogenic warming (e.g. refs. 93, 97–101).

Monthly climate change deltas are calculated for the following variables: geopotential height, relative humidity, specific humidity, surface temperature, atmospheric temperature, horizontal winds, surface pressure, mean sea level pressure, and sea ice concentration. The historical climate delta is calculated by subtracting the mean 30-year climate for March and April in the period 1985–2014 from the mean 30-year climate for March and April respectively in the period 1885–1914. This delta approximates the recent climate change in the dynamic and thermodynamic environment over the last century, i.e., from pre-industrial times to the present. Adding this delta to the current climate conditions essentially “removes” the influence of recent climate change on the Arctic environment. Similarly, the future climate delta is calculated by subtracting the mean 30-year climate for March (April) in 1985–2014 from the mean 30-year climate for March (April) in 2070–2099. This delta represents the average change projected in the large-scale environment from the present day to the end of the 21st century.

Studies have shown that results from experiments such as these may be sensitive to the choice of delta applied. Trapp et al.^102^ found that variability in the PGW response is lost when using a multi-model average delta compared to individual deltas from GCM members, and results from simulations with 10-year deltas did not consistently differ from those with 30-year deltas. However, other studies e.g.^103,104^ discuss that individual GCM model runs may contain large internal variability and may be unrepresentative of the forced climate change at the decadal scale. Using a multi-model ensemble average minimizes the influence of unforced climate variations, model errors, and the large range of modeled climate sensitivity to greenhouse forcings^104^. This study considers that using multi-model, 30-year average climatology to calculate the climate delta is appropriate for this application in the Arctic where future projections remain uncertain and varied between models and large decadal oscillations (e.g. the Arctic Oscillation) play a major role in governing atmospheric conditions in any given year.

The 30-year climatologies are calculated from 8 different model realizations from 6 modeling centers that participated in the Climate Model Intercomparison Project (CMIP6^65^, see Supplementary Table 1). The selection was based on data available at the time of the delta calculation, grid consistency, and the type of experiment conducted. Some instances (e.g., CNRM-ESM2-1 model) did not include information on sea ice concentration while other instances (e.g., IPSL-CM6A-LR and CNRM-CM6-1) had missing data at specific points on the global grid and therefore could not be used for this study. The 30-year historical, current, and future averages are calculated from the CMIP6 historical runs and future climate simulations under the shared socioeconomic pathway 8.5 (SSP5-8.5^105^) scenario. This collection of climate realizations estimates that global near-surface (2 m) air temperatures have increased by ~0.61 °C from the historical (1885–1914) to current (1985–2014) climate, and predict a further increase of ~3.22 °C from the current to future (2070–2099) climate following the SSP5-8.5 scenario (see Supplementary Table 1). The SSP5-8.5 scenario assumes that anthropogenic activity will increase at the same rate as observed in the past few decades. While it is debated in the literature that this scenario may not be the most likely to occur^106^, SSP5–8.5 is the highest and most extreme emissions scenario considered in CMIP6. Therefore, this study uses this scenario to explore the response of cyclone characteristics to the largest projected changes in the local and global climate.

The PGW approach used in this study has implicit assumptions, and results should be interpreted accordingly. In each climate scenario, a cyclone vortex structure, as given by present-day ERA5 reanalysis data, is ‘seeded’ within the domain at the start of the simulation. The dynamic and thermodynamic changes for historical and future climate change are applied to the environment where the cyclone is in the early stages of development. This study, therefore, does not explore or analyze changes in the likelihood of Arctic cyclone genesis or frequency with climate change. This method allows us to examine the extent to which recent climate change has altered Arctic cyclone intensification and characteristics, and how future projected climate change may continue to affect cyclone behavior in the Arctic.

### Cyclone case studies

Possible cyclone cases occurring between 2000 and 2021 were first identified by applying the Melbourne University cyclone finding and tracking scheme^47,53,107^ to ERA5 SLP data. The final cases for this study were chosen based on: (1) timing in the year—cases that occurred in spring when interactions between the atmosphere and sea ice have important implications for sea ice survivability during the melt season; (2) geographic distribution—cases that provided a variety of trajectories across the Arctic within the WRF domain (Fig. S1); and (3) intensity—cases that reached a minimum SLP of 980 hPa or lower in the current climate with the main intensification period occurring in the model domain. This ensured that a defined and closed system could be simulated in the model setup. From the iterative analysis, nine Arctic cyclones were chosen for this study. Six occurred in March (Cyclones A–C, G–I, Table 2, Supplementary Table 2) and were perturbed with average March historical and future climate deltas (Fig. 1; Supplementary Fig. 3), and three occurred in April (Cyclones D–F, Supplementary Table 2) and were perturbed with April climate deltas (Supplementary Fig. 2). Three of the cyclone cases (A–C from 2020) were chosen because of their possible coincidence with the trajectory of the drifting MOSAiC expedition. Surface pressure, wind speed, and air temperature observations from both the weather station on board the Polarstern and on the sea ice as part of the observing network of MOSAiC^62^ were examined to confirm the detection of a cyclone event in the vicinity of the ship. These cases present a unique opportunity for detailed validation of the WRF cyclone simulations with in-situ observational data and serve as the primary cases presented in this paper.

Cyclone A formed ~11-March-2020 UTC south of Svalbard and traveled poleward through the Barents Sea towards the Central Arctic before tracking westward and back south towards Svalbard, decaying by ~15-March-2020 UTC. Cyclone B formed ~18-March-2020 UTC in the East Siberian Sea and traveled poleward and westward through the Central Arctic before traveling southward towards Severnaya Zemlya and decaying near the Russian coast ~21-March-2020 UTC. Cyclone C formed ~30-March-2020 UTC near the New Siberian Islands and traveled poleward into the Central Arctic and then southward and westward towards Franz Josef Land and decaying ~3-April-2020 UTC. We note that recent work has demonstrated that cyclone counts coinciding with the MOSAiC expedition were near or above the 75th percentile in February and March 2020 relative to the 1979–2020 monthly median and cyclones in February to April were stronger than average^108^.

### Model evaluation

At present, there are no direct observations and records tracking cyclone activity and characteristics in the Arctic. Therefore, the WRF model simulations are evaluated by comparing the atmospheric pressure and temperature in the current climate simulations to meteorological data recorded during the MOSAiC expedition^109^. In situ MOSAiC measurements are averaged to hourly resolution and comparisons are made using the model latitude and longitude grid box closest to the recorded location of the observing network at that time step (Supplementary Fig. 9). Minimum sea level pressure (SLP) and air-surface temperature gradients are critical drivers and characteristics of cyclone activity. Supplementary Fig. 9a, c, e shows that the WRF simulated SLP is in excellent agreement with the observed values over all three simulations. The model is also able to represent the relative magnitudes and changes in near-surface (2 m) air temperatures (Supplementary Fig. 9b, d, f). The discrepancies between the model and observed temperatures (average +3.12, −0.55, +2.09 °C for cases A–C, respectively) may be due to deviations in the elevation of the simulated and retrieved temperatures and differences in resolution between the in-situ point measurement and temperatures averaged over a 4 km grid box.

Overall, the assessment demonstrates that the model configuration developed for this study can accurately represent atmospheric variables and their change over time. Discrepancies are within reason given differences in resolution and possible elevation and instrument errors with in-situ retrievals. We, therefore, consider that the high-resolution WRF regional climate configuration can provide a sufficient representation of the intensification and characteristics of Arctic cyclones in the current climate to assess their relative change in response to recent and future climate change.

## Supplementary information

Supplementary Information

## Data Availability

ERA5 reanalysis data used for cyclone tracking to identify cases and the WRF simulation boundary and initial conditions can be accessed here: https://cds.climate.copernicus.eu/cdsapp#!/home. CMIP6 model results used for calculating the climate delta can be accessed here: https://esgf-node.llnl.gov/search/cmip6/. MOSAiC Data used in this manuscript was collected from the Atmospheric Radiation Measurement (ARM) User Facility, a U.S. Department of Energy (DOE) Office of Science User Facility managed by the Biological and Environmental Research Program, under expedition number MOSAiC20192020 and project identifier AWI_PS122_00. Meteorological Measurements associated with the Surface Meteorological Instrumentation (PWD) and Aerosol Observing System (AOSMET). 2020-03-01 to 2020-04-03, ARM Mobile Facility (MOS) MOSAiC (Drifting Obs - Study of Arctic Climate); AMF2 (M1). Compiled by J. Kyrouac, S. Springston, and D. Holdridge. ARM Data Center. Data set accessed 2021-05-27 at 10.5439/1025153. The WRF simulation data generated and used for this analysis have been deposited in the Zenodo database under accession codes: 10.5281/zenodo.7131284; 10.5281/zenodo.7131287; 10.5281/zenodo.7126117.

## Code availability

The Melbourne University cyclone tracking and finding a scheme that was used to identify possible cyclone cases for simulation case be accessed here: https://cyclonetracker.earthsci.unimelb.edu.au/.

WRF v3.9.1.1 source code can be accessed here: https://github.com/NCAR/WRFV3/releases.

Specific WRF namelist files for experiment set up in this study can be accessed here: 10.5281/zenodo.7131284.

Climate Data Operator Software used to calculate the climate deltas can be accessed here: 10.5281/zenodo.5614769.
