# Supplementary material for: The influence of recent and future climate change on spring Arctic cyclones
Source: Nat Commun. 2022 Nov 9;13:6514. doi: 10.1038/s41467-022-34126-7 (PMC9646868; doi:10.1038/s41467-022-34126-7)
Supplement: Supplementary file 1 — Supplementary Information [file 41467_2022_34126_MOESM1_ESM.pdf]

The Influence of Recent and Future Climate Change on Spring Arctic Cyclones

Supplementary Information

Chelsea L. Parker[1,2], Priscilla A. Mooney[3], Melinda A. Webster[4], and Linette N. Boisvert[1]

[1]Cryospheric Sciences Lab, NASA Goddard Space Flight Center, Greenbelt, Maryland 20771, U.S.A.
[2]ESSIC, University of Maryland, College Park, Maryland 20740, U.S.A.
[3]NORCE, Bjerknes Centre for Climate Research, Jahnebakken 5, 5007, Bergen, Norway
[4]University of Alaska Fairbanks, Geophysical Institute, Fairbanks, Alaska, 99775, U.S.A.

Affiliations

[1]Cryospheric Sciences Lab, NASA Goddard Space Flight Center, Greenbelt, Maryland 20771, U.S.A.
[2]ESSIC, University of Maryland, College Park, Maryland 20740, U.S.A.
[3]NORCE Norwegian Research Centre, Bjerknes Centre for Climate Research, Jahnebakken 5, 5007, Bergen, Norway
[4]University of Alaska Fairbanks, Geophysical Institute, Fairbanks, Alaska, 99775, U.S.A.

*Corresponding Author: Chelsea L Parker, Chelsea.L.Parker@NASA.gov, cparker0@umd.edu

Supplementary materials to manuscript submitted to
Nature Communications on September 30th 2022

Supplementary Tables

Model Name	Realization	Historical 1885-1914 (°C)	Current 1985-2014 (°C)	Future 2070-2099 (°C)	Temp Change Current - Historical (°C)	Temp Change Future - Current (°C)
BCC-CSM2-MR	r1i1p1f1	14.72	15.27	18.49	0.55	3.22
CAMS-CSM1-0	r1i1p1f1	13.83	14.43	16.77	0.6	2.34
CAMS-CSM1-0	r2i1p1f1	13.79	14.38	16.71	0.59	2.28
EC-EARTH3	r1i1p1f1	13.85	14.6	18.78	0.75	4.18
MIROC6	r1i1p1f1	15.22	15.75	18.72	0.53	2.97
MIROC6	r2i1p1f1	15.24	15.75	18.72	0.51	2.97
MRI-ESM2-0	r1i1p1f1	13.78	14.32	17.80	0.54	3.48
CESM2	r10i1p1f1	14.03	14.77	19.08	0.74	4.31
MME AVERAGE	-	14.30	14.91	18.13	0.61	3.22

Supplementary Table 1: The CMIP6 ensemble members used to calculate the climate change deltas for the high-resolution regional simulations.
Details of the Climate Model Intercomparison Project (CMIP6) modeling centers and their ensemble members used to calculate the average historical and future climate deltas for the simulations in this study. The global average near surface (2m) temperature for the historical (1885-1914), current (1985-2014), and future (2070-2099) time periods and the difference between the time periods is presented for context.

Run Name	Initialization Time (UTC)	Total Integration time (hours)	Climate Conditions	CO_2 / CH_4 / N_2O Concentration (ppm/ppb/ppb)
CycloneD_CC	2020-04-19 12:00	66	ERA5 current climate	379 / 1774 / 319
CycloneE_CC	2014-04-11 06:00	102	ERA5 current climate	379 / 1774 / 319
CycloneF_CC	2014-04-16 12:00	102	ERA5 current climate	379 / 1774 / 319
CycloneG_CC	2017-03-17 18:00	66	ERA5 current climate	379 / 1774 / 319
CycloneH_CC	2011-03-19 18:00	78	ERA5 current climate	379 / 1774 / 319
CycloneI_CC	2021-03-08 18:00	102	ERA5 current climate	379 / 1774 / 319
CycloneD_HC	2020-04-19 12:00	66	ERA5 current climate + ΔHC	297 / 895 / 280
CycloneE_HC	2014-04-11 06:00	102	ERA5 current climate + ΔHC	297 / 895 / 280
CycloneF_HC	2014-04-16 12:00	102	ERA5 current climate + ΔHC	297 / 895 / 280
CycloneG_HC	2017-03-17 18:00	66	ERA5 current climate + ΔHC	297 / 895 / 280
CycloneH_HC	2011-03-19 18:00	78	ERA5 current climate + ΔHC	297 / 895 / 280
CycloneI_HC	2021-03-08 18:00	102	ERA5 current climate + ΔHC	297 / 895 / 280
CycloneD_FC	2020-04-19 12:00	66	ERA5 current climate + ΔFC	975 / 2595 / 385
CycloneE_FC	2014-04-11 06:00	102	ERA5 current climate + ΔFC	975 / 2595 / 385
CycloneF_FC	2014-04-16 12:00	102	ERA5 current climate + ΔFC	975 / 2595 / 385
CycloneG_FC	2017-03-17 18:00	66	ERA5 current climate + ΔFC	975 / 2595 / 385
CycloneH_FC	2011-03-19 18:00	78	ERA5 current climate + ΔFC	975 / 2595 / 385
CycloneI_FC	2021-03-08 18:00	102	ERA5 current climate + ΔFC	975 / 2595 / 385

Supplementary Table 2: Experimental set-up for the model simulations of cyclone case studies D-I under different climate forcings.
Details of the initialization time, duration, boundary conditions, and prescribed concentration of greenhouse gases for each of the cyclone case studies (D-I) and climate scenarios: current climate (CC), historical climate (HC), future climate (FC).

Change in Characteristic	Cyclone D CC-HC	Cyclone E CC-HC	Cyclone F CC-HC	Cyclone D FC-CC	Cyclone E FC-CC	Cyclone F FC-CC
Average Sea ice concentration (%)	Max: -14.93 Avg: -0.49	Max: -1.13 Avg: -0.07	Max: -14.40 Avg: -1.34	Max: -54.68 Avg: -32.07	Max: - 11.21 Avg: -3.67	Max: -53.93 Avg: -18.72
Average Surface temperature (°C)	Max: 4.42 Avg: 1.82	Max: 2.91 Avg: 1.33	Max: 2.55 Avg: 1.29	Max: 15.01 Avg: 7.41	Max: 12.37 Avg: 8.83	Max: 14.06 Avg: 10.32
Minimum Sea level pressure (hPa)	Max: -2.43 Avg: -1.39	Max: 0.31 Avg: 0.66	Max: -1.03 Avg: 0.23	Max: -6.13 Avg: -4.01	Max: -6.39 Avg: -3.47	Max: -11.38 Avg: -6.19
% Lifecycle at maximum intensity	-3.08	0	-0.98	4.62	3.88	8.82
Maximum 10m wind speed (ms^{-1})	Max: 4.32 Avg: 0.22	Max: 2.43 Avg: 0.14	Max: 2.31 Avg: -0.38	Max: 7.58 Avg: 1.96	Max: 4.39 Avg: 1.45	Max: 7.86 Avg: 2.85
Average Sensible heat flux (Wm^{-2})	Max: 13.89 Avg: 0.21	Max: 5.33 Avg: 0.43	Max: 17.77 Avg: 0.97	Max: 68.88 Avg: 20.93	Max: 15.64 Avg: 5.14	Max: 68.03 Avg: 10.59
Average Latent heat flux (Wm^{-2})	Max: 6.46 Avg: 0.59	Max: 1.81 Avg: 0.11	Max: 20.16 Avg: 1.34	Max: 91.24 Avg: 41.18	Max: 8.78 Avg: 4.17	Max: 85.54 Avg: 30.75
Average 2m temp - surface temp	Max: -1.43 Avg: -0.06	Max: -0.61 Avg: -0.07	Max: -0.68 Avg: -0.04	Max: -2.10 Avg: -0.78	Max: -1.11 Avg: -0.48	Max: -1.87 Avg: -0.48
Average CAPE ($J kg^{-1}$)	Max: 9.48 Avg: 0.42	Max: 0 Avg: 0	Max: 12.07 Avg: 0.69	Max: 79.76 Avg: 15.10	Max: 7.59 Avg: 0.34	Max: 53.10 Avg: 10.88
Average 2m temp (°C)	Max: 4.18 Avg: 1.75	Max: 2.30 Avg: 1.26	Max: 2.39 Avg: 1.25	Max: 13.21 Avg: 6.58	Max: 11.55 Avg: 8.35	Max: 13.61 Avg: 9.84
Sum snowfall in 200km area ($mm h^{-1}$)	Max: 185.48 Avg: -5.58	Max: 109.55 Avg: 11.95	Max: 133.18 Avg: -55.97	Max: 952.22 Avg: -18.56	Max: 503.84 Avg: 151.20	Max: 367.94 Avg: - 184.08
Sum rainfall in 200km area ($mm h^{-1}$)	Max: 3.85 Avg: 0.24	Max: 1.31 Avg: 0.13	Max: 601.78 Avg: 31.50	Max: 887.38 Avg: 220.25	Max: 77.08 Avg: 7.54	Max: 3062.20 Avg: 549.26
Median radius (km)	Max: 170.96 Avg: 8.33	Max: 134.39 Avg: -15.57	Max: 257.48 Avg: 7.18	Max: 363.73 Avg: 10.92	Max: 199.08 Avg: -3.40	Max: 239.29 Avg: -48.92

Supplementary Table 3: Numerical summary of changes in simulated environmental and cyclone (D-F) characteristics from historical and future climate perturbation.
The average and maximum difference in the surface, atmospheric, near surface flux, and cyclone variables of interest over the length of the simulations. Differences are calculated as (current climate (CC) - historical climate (HC)) and (future climate (FC) - current climate (CC)) simulation at each timestep for each cyclone case D-F.

Change in Characteristic	Cyclone G CC-HC	Cyclone H CC-HC	Cyclone I CC-HC	Cyclone G FC-CC	Cyclone H FC-CC	Cyclone I FC-CC
Average Sea ice concentration (%)	Max: -31.90 Avg: -4.07	Max: -4.43 Avg: -0.10	Max: -0.66 Avg: -0.06	Max: -55.72 Avg: -9.65	Max: -48.37 Avg: -13.36	Max: -22.92 Avg: -11.36
Average Surface temperature (°C)	Max: 3.56 Avg: 1.91	Max: 4.33 Avg: 1.87	Max: 2.97 Avg: 1.15	Max: 18.13 Avg: 12.61	Max: 19.81 Avg: 13.66	Max: 26.73 Avg: 15.70
Minimum Sea level pressure (hPa)	Max: -2.03 Avg: -1.24	Max: -0.50 Avg: 0.93	Max: -2.84 Avg: -0.32	Max: -28.49 Avg: -11.68	Max: -8.31 Avg: -4.54	Max: -11.51 Avg: -6.17
% Lifecycle at maximum intensity	1.49	8.86	3.57	1.49	-15.19	30.95
Maximum 10m wind speed (ms^{-1})	Max: 6.19 Avg: 0.97	Max: 3.89 Avg: -0.05	Max: 4.03 Avg: 0.11	Max: 9.75 Avg: 4.09	Max: 8.74 Avg: 4.18	Max: 9.35 Avg: 4.76
Average Sensible heat flux (Wm^{-2})	Max: 39.67 Avg: 4.31	Max: 8.19 Avg: -0.24	Max: 4.60 Avg: -0.40	Max: 143.45 Avg: 35.67	Max: 76.62 Avg: 29.12	Max: 66.11 Avg: 28.80
Average Latent heat flux (Wm^{-2})	Max: 35.62 Avg: 3.64	Max: 2.24 Avg: -0.31	Max: 1.68 Avg: -0.10	Max: 214.06 Avg: 75.29	Max: 83.42 Avg: 28.78	Max: 56.37 Avg: 25.15
Average 2m temp - surface temp	Max: -1.55 Avg: -0.26	Max: -0.60 Avg: 0.07	Max: -0.29 Avg: 0.09	Max: -3.76 Avg: -1.88	Max: -4.45 Avg: -1.66	Max: -5.69 Avg: -2.26
Average CAPE (J kg^{-1})	Max: 4.01 Avg: -0.76	Max: 0 Avg: 0	Max: 0 Avg: 0	Max: 256.67 Avg: 43.03	Max: 29.10 Avg: 5.87	Max: 37.21 Avg: 9.80
Average 2m temp (°C)	Max: 3.38 Avg: 1.65	Max: 4.55 Avg: 1.94	Max: 2.82 Avg: 1.24	Max: 15.82 Avg: 10.73	Max: 17.04 Avg: 12.00	Max: 21.48 Avg: 13.45
Sum snowfall (mm h^{-1})	Max: 420.93 Avg: 45.56	Max: 107.69 Avg: -11.01	Max: 35.98 Avg: 2.80	Max: 1661.35 Avg: 94.14	Max: 533.59 Avg: 125.48	Max: 322.24 Avg: 137.36
Sum rainfall (mm h^{-1})	Max: 319.12 Avg: 18.29	Max: 2.01 Avg: 0.18	Max: 0 Avg: 0	Max: 2507.17 Avg: 845.62	Max: 99.53 Avg: 12.72	Max: 0.98 Avg: 0.08
Median radius (km)	Max: 180.60 Avg: -9.42	Max: 312.31 Avg: -5.31	Max: 237.97 Avg: -7.89	Max: 330.97 Avg: 28.48	Max: 216.67 Avg: -100.42	Max: 256.86 Avg: -7.70

Supplementary Table 4: Numerical summary of changes in simulated environmental and cyclone (G-I) characteristics from historical and future climate perturbation.

The average and maximum difference in the surface, atmospheric, near surface flux, and cyclone variables of interest over the length of the simulations. Differences are calculated as (current climate (CC) - historical climate (HC)) and (future climate (FC) - current climate (CC)) simulation at each timestep for each cyclone case G-I. Differences for Cyclone I are calculated from timestep 1-91, up to where the current climate simulation interacts with the land (see Supplementary Figure 5c, 7l).

Supplementary Figures

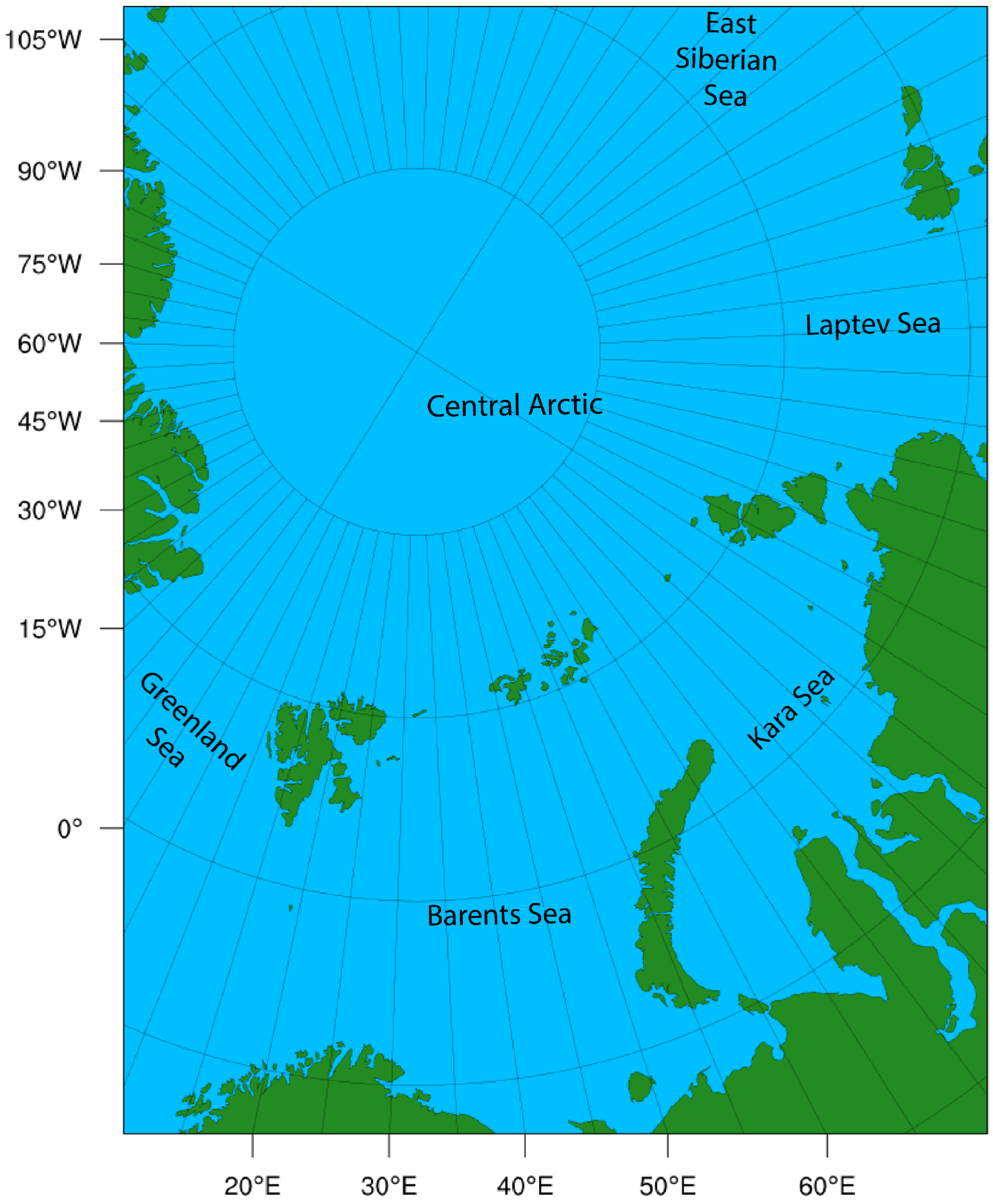

Supplementary Figure 1: WRF regional model domain configuration.
The domain configuration of the Weather Research and Forecasting (WRF) regional model. The model is configured with 4km horizontal grid spacing and 51 vertical levels. Particular areas of interest for the analysis are labeled.

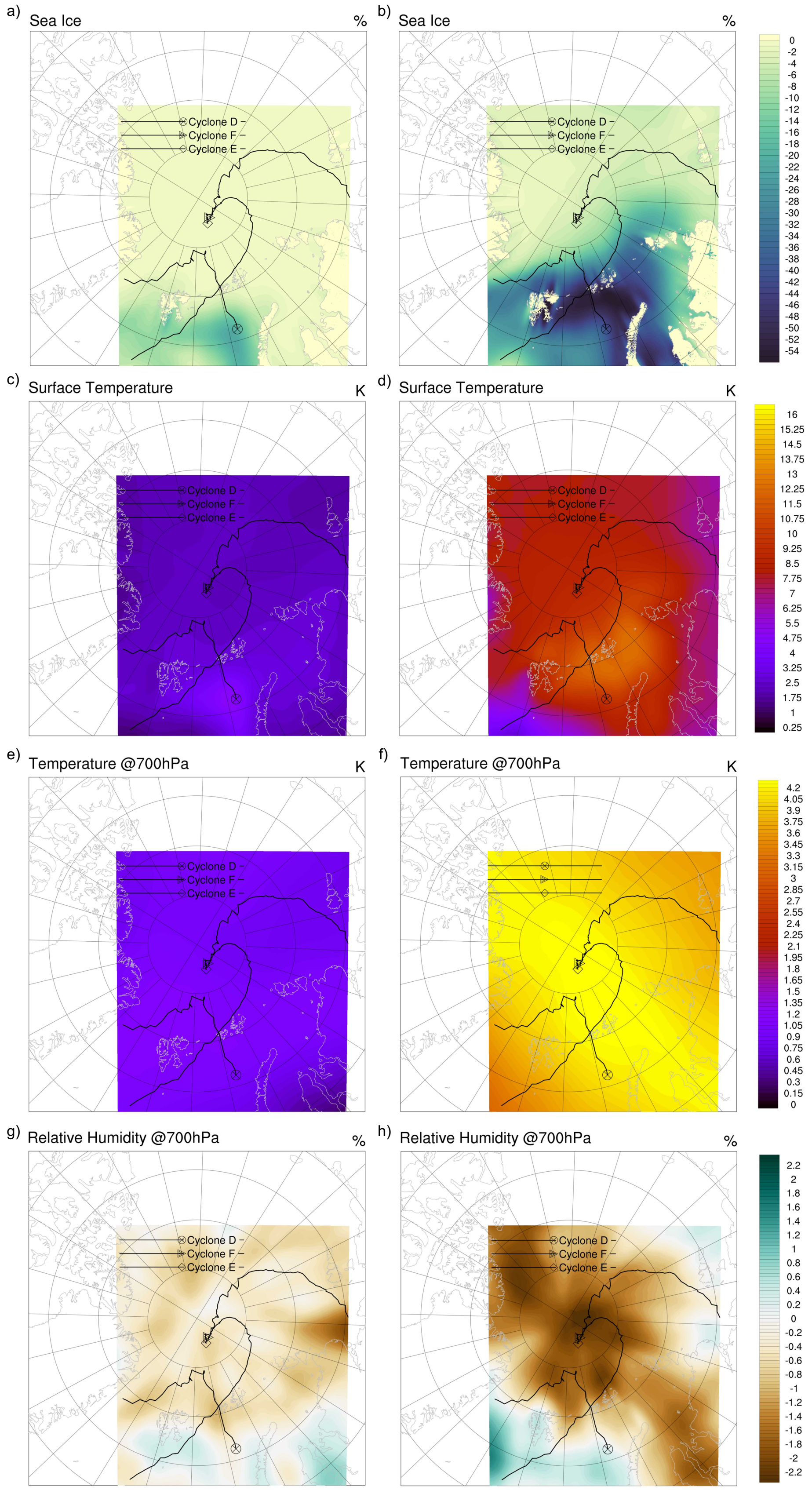

Supplementary Figure 2: The historical and future April climate deltas calculated from Climate Model Intercomparison Project 6 results.

The calculated difference in April sea ice concentration, surface temperature, and temperature and relative humidity at 700hPa for the average current (1985-2014) minus the average historical (1886-1915) climate (panels **a, c, e, g** respectively) and the average future (2070-2100) minus the average current climate (panels **b, d, f, h**). The simulated tracks of cyclone cases D,E,F in the current climate are overlain. Markers show the end of the trajectories.

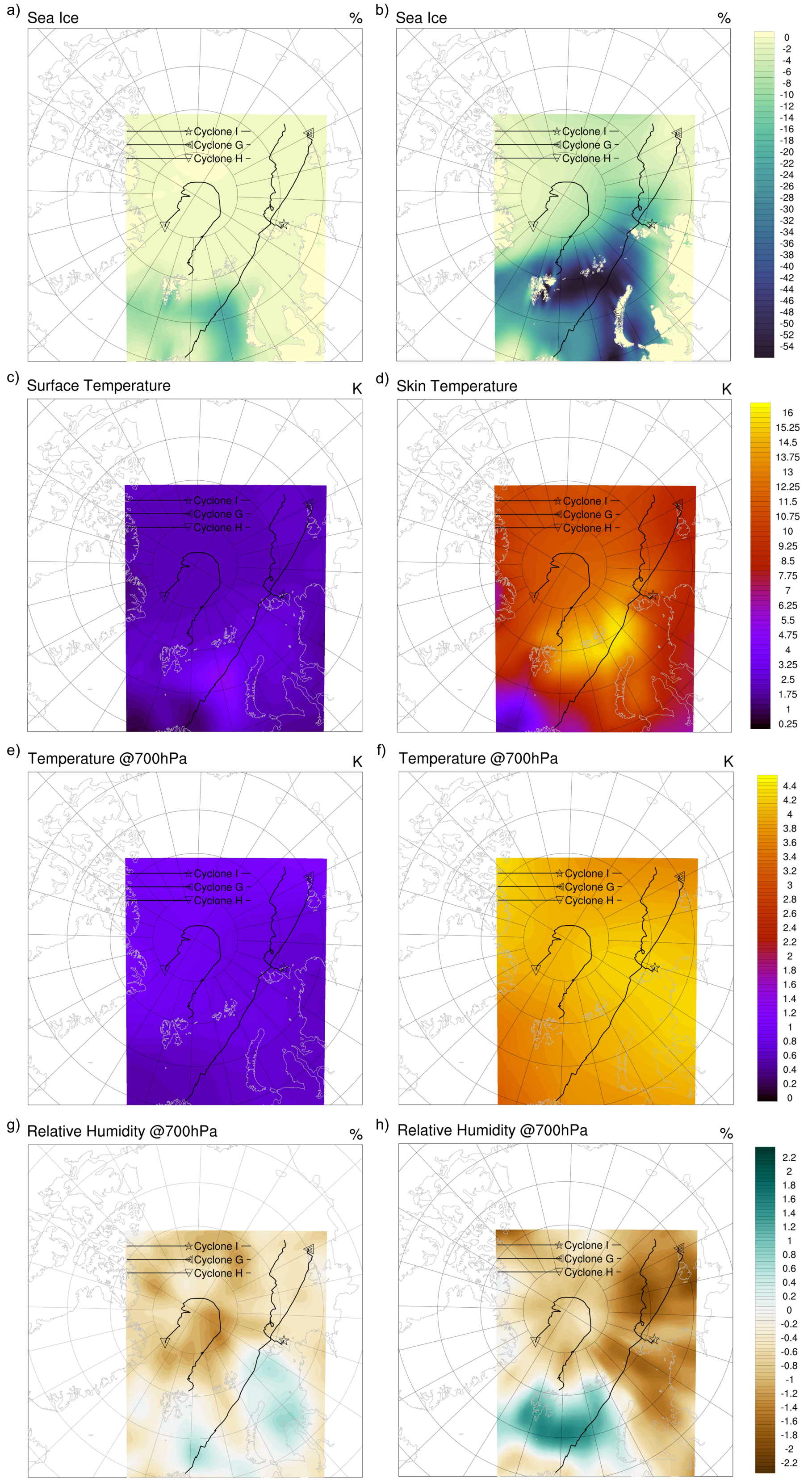

Supplementary Figure 3: The historical and future March climate deltas calculated from Climate Model Intercomparison Project 6 results.

The calculated difference in the March sea ice concentration, surface temperature, and temperature and relative humidity at the 700hPa level of the atmosphere for the average current (1985-2014) minus the average historical (1886-1915) climate (panels **a, c, e, g** respectively) and the average future (2070-2100) minus the average current climate (panels **b, d, f, h**). The simulated tracks of the cyclone cases G,H,I in the current climate are overlain. Markers show the end of the trajectories.

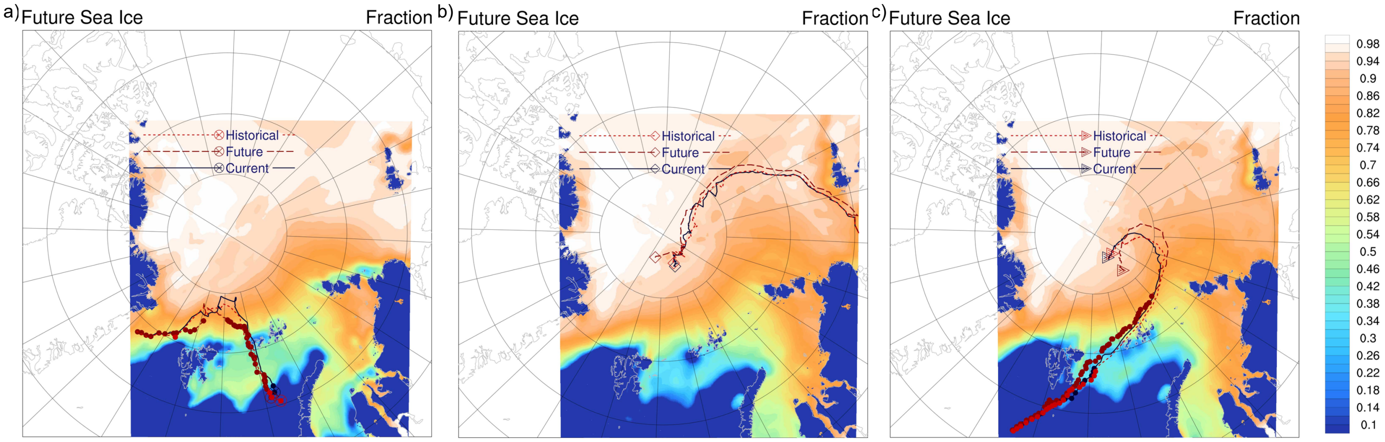

Supplementary Figure 4: Alterations in April cyclone trajectories in response to climate perturbations. Panels **a,b,c** show the average sea ice concentration (fraction) over the future climate simulations of Cyclones D,E,F respectively with the simulated cyclone tracks from historical, current, and future climate simulations overlain. Open markers show the end of the trajectories, filled markers indicate where average sea ice concentration beneath the cyclone is ≤75%.

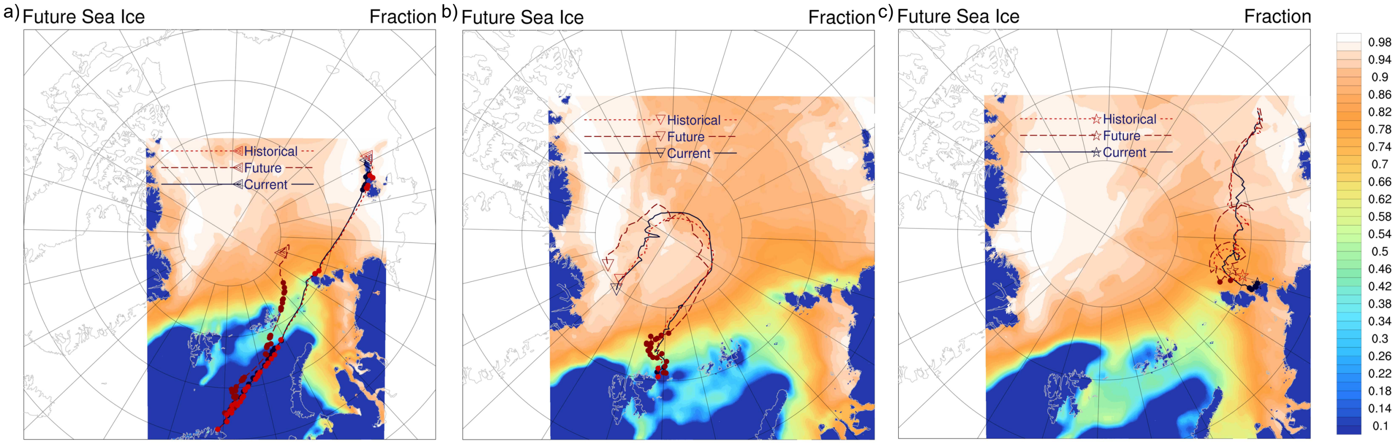

Supplementary Figure 5: Alterations in March cyclone trajectories in response to climate perturbations. Panels **a,b,c** show the average sea ice concentration (fraction) over the future climate simulations of Cyclones G,H,I respectively with the simulated cyclone tracks from historical, current, and future climate simulations overlain. Open markers show the end of the trajectories, filled markers indicate where average sea ice concentration beneath the cyclone is ≤75%.

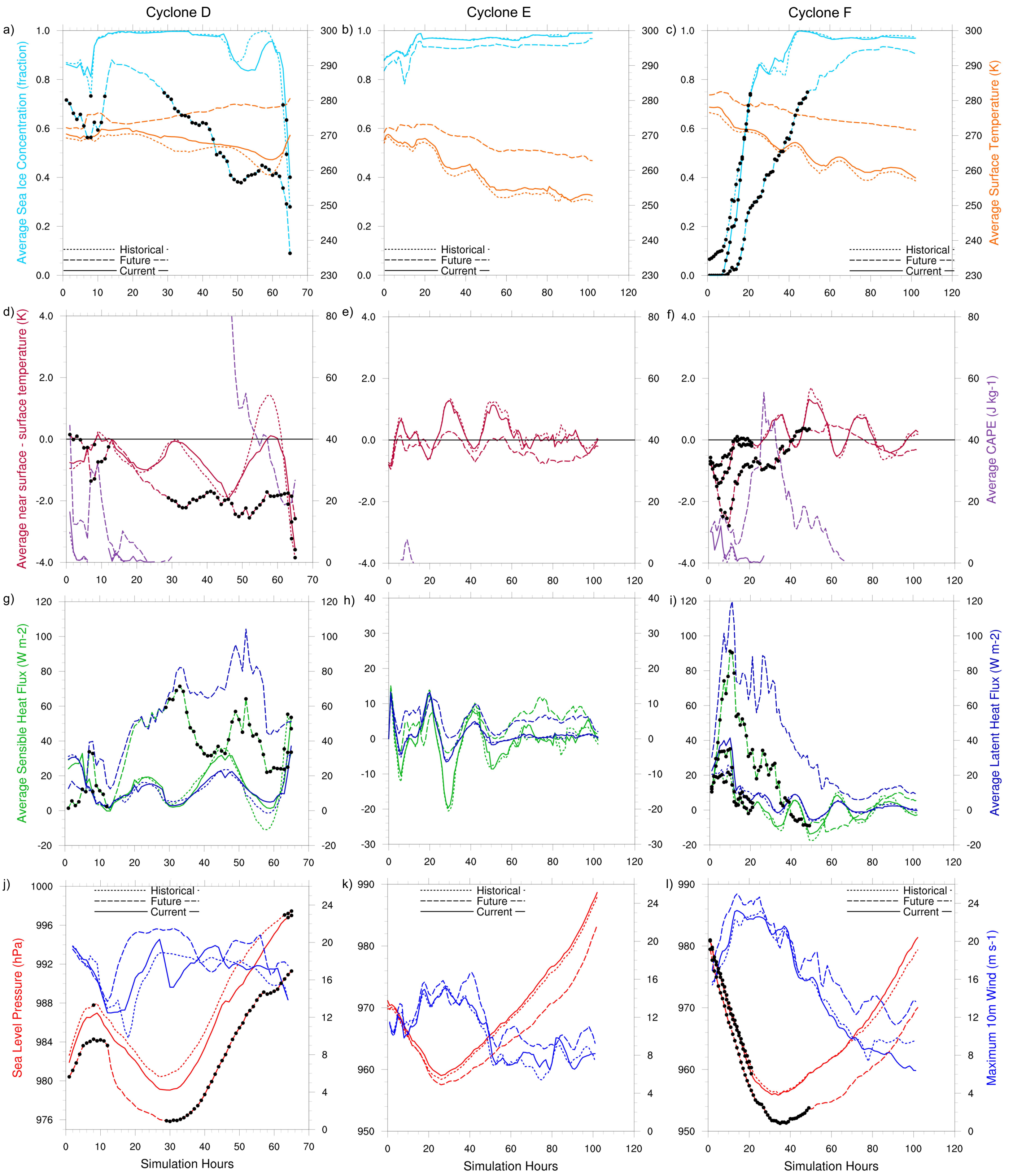

Supplementary Figure 6: The evolution of surface and thermodynamic conditions and cyclone intensity over the April cyclone lifecycles in the contrasting climates.

The hourly sea ice concentration (fraction) and surface temperature (K) (a-c), difference in near surface air temperature and surface temperature (K) and Convective Available Potential Energy (J kg^{-1}) (d-f), sensible and latent heat fluxes (W m^{-2}) (g-i) averaged over a 200km^2 area around the cyclone center point throughout the simulations of Cyclones D, E, F. Panels j-l show the sea level pressure (hPa) at the center-point of the cyclone and the maximum 10m wind speed in a 200km2 area around the storm center. The black markers indicate time steps where the average sea ice concentration in the same area is ≤75%.

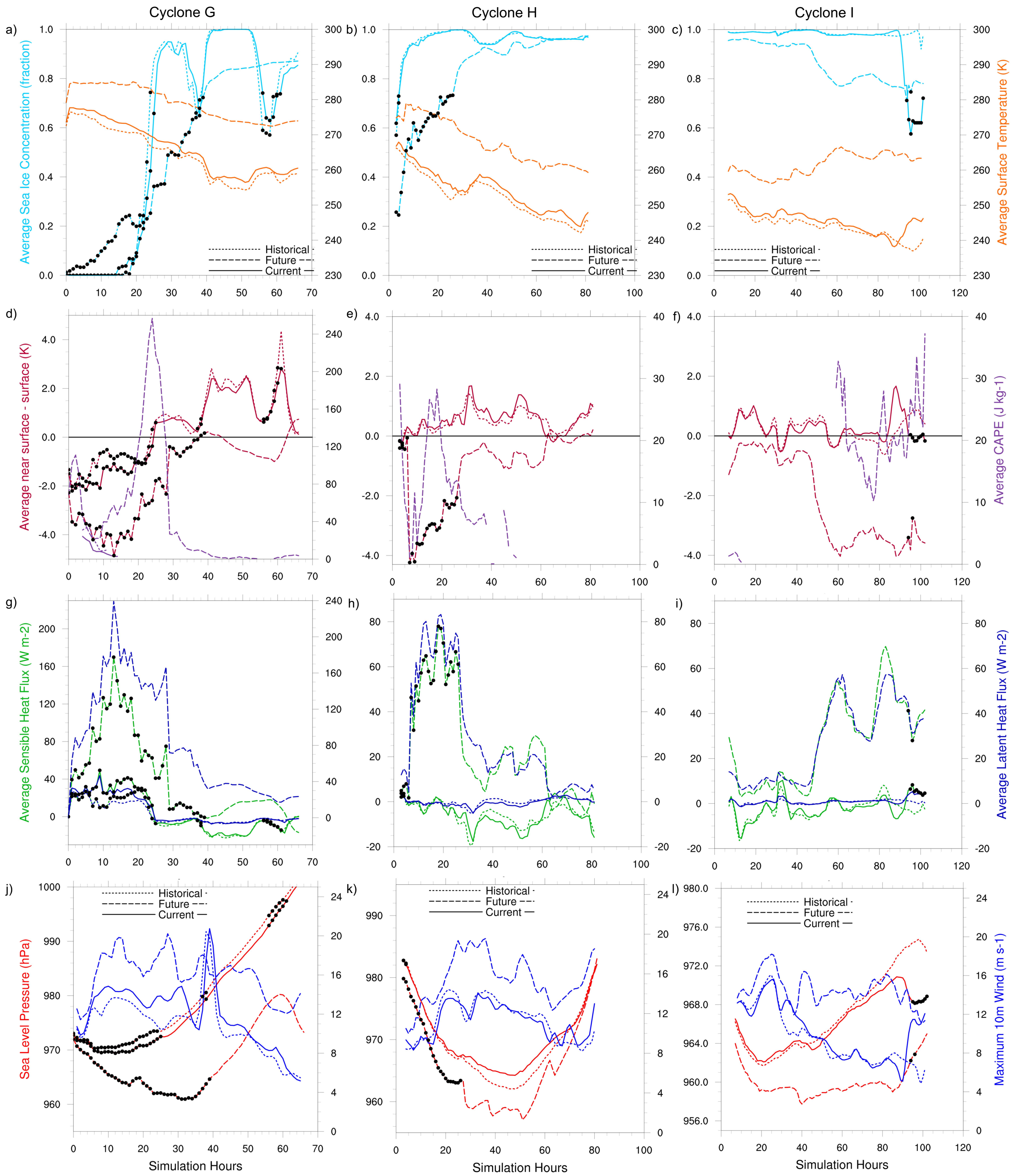

Supplementary Figure 7: The evolution of surface and thermodynamic conditions and cyclone intensity over the March cyclone lifecycles in the contrasting climates.

The hourly sea ice concentration (fraction) and surface temperature (K) (a-c), difference in near surface air temperature and surface temperature (K) and Convective Available Potential Energy (J kg-1) (d-f), sensible and latent heat fluxes (W m^{-2}) (g-i) averaged over a 200km^2 area around the cyclone center point throughout the simulations of Cyclones G, H, I. Panels j-l show the sea level pressure (hPa) at the center-point of the cyclone and the maximum 10m wind speed in a 200km^2 area around the storm center. The black markers indicate time steps where the average sea ice concentration in the same area is ≤75%.

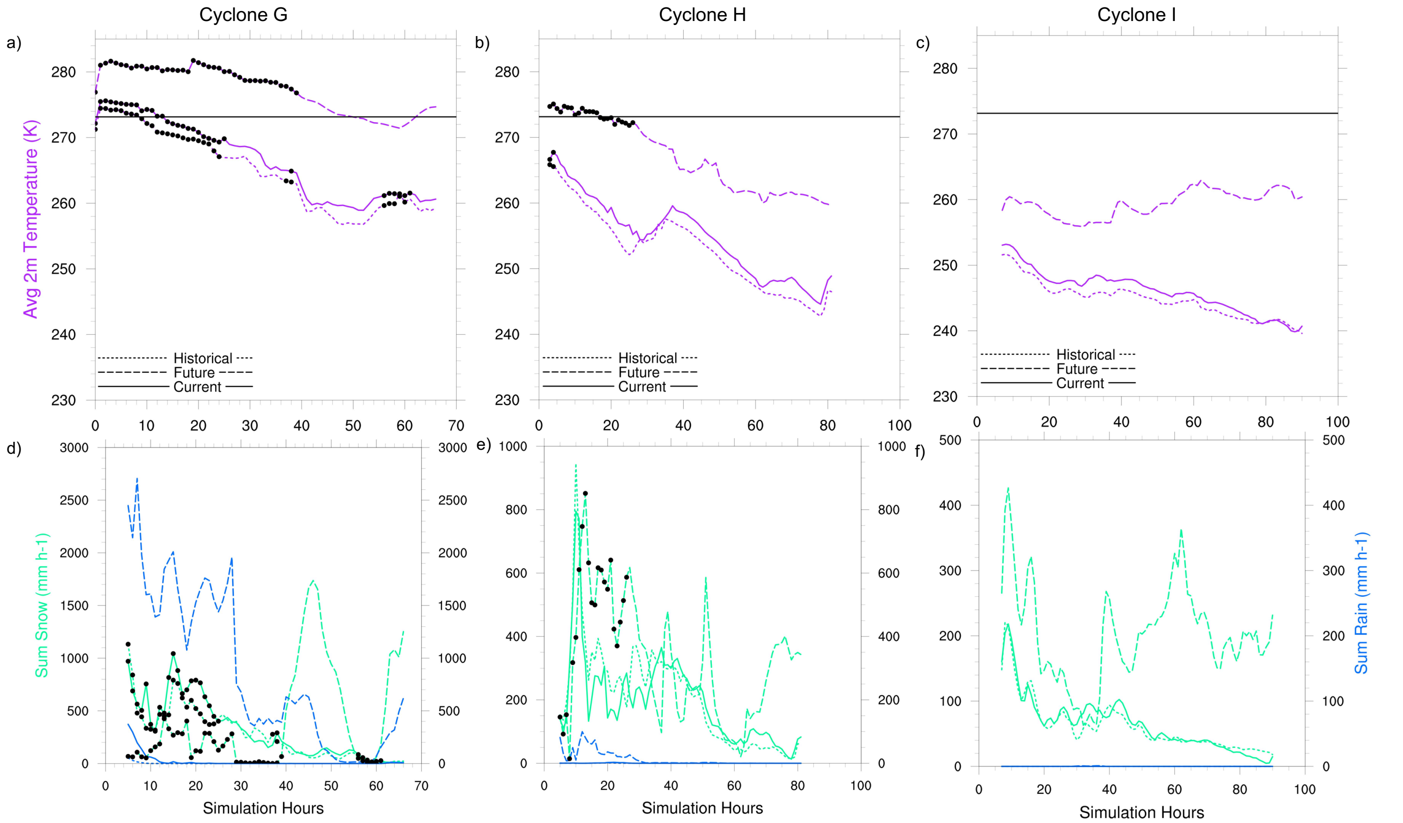

Supplementary Figure 8: The evolution of near surface air temperature and precipitation (snow and rain) rates over March cyclone lifecycles in the contrasting climates.

The hourly cyclone near surface (2m) temperature (K, panels **a,b,c**) averaged and snowfall and rainfall rates (mm h^{-1} panels **d,e,f**) summed and the over a 200km^2 area around the cyclone center throughout the simulations for March Cyclones G, H, I. The black markers indicate time steps where the average sea ice concentration in the same area is ≤75%. The solid line in panels a,b,c marks the melting/freezing point (0°C).

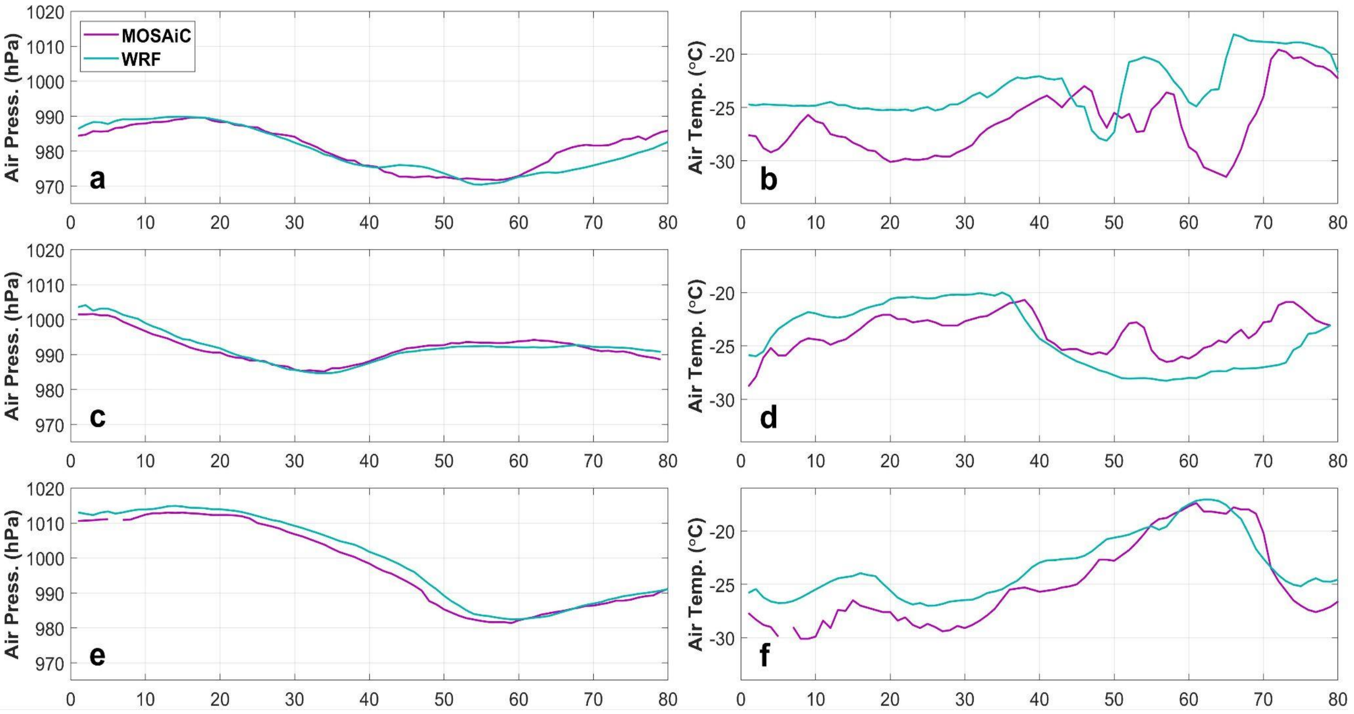

Supplementary Figure 9: Validation of WRF simulated sea level pressure and near surface air temperature with MOSAiC observations.

A comparison of sea level air pressure (hPa) and near surface (2m) air temperatures (°C) between the MOSAiC observations and WRF simulations for Cyclones A (a-b), B (c-d), and C (e-f). *In-situ* measurements are averaged to hourly temporal resolution and compared to simulated values from the grid box closest to the recorded latitude/longitude of the observing network at that time step.
